# Experimentally simulating the dynamics of quantum light and matter at deep-strong coupling

**DOI:** 10.1038/s41467-017-01061-x

**Published:** 2017-11-23

**Authors:** N. K. Langford, R. Sagastizabal, M. Kounalakis, C. Dickel, A. Bruno, F. Luthi, D. J. Thoen, A. Endo, L. DiCarlo

**Affiliations:** 1 QuTech, Delft University of Technology, Lorentzweg 1, 2628 CJ Delft, The Netherlands; 2 Kavli Institute of Nanoscience, Delft University of Technology, Lorentzweg 1, 2628 CJ Delft, The Netherlands; 3 Department of Microelectronics, Faculty of Electrical Engineering, Mathematics and Computer Science, Delft University of Technology, Mekelweg 4, 2628 CD Delft, The Netherlands

## Abstract

The quantum Rabi model describing the fundamental interaction between light and matter is a cornerstone of quantum physics. It predicts exotic phenomena like quantum phase transitions and ground-state entanglement in ultrastrong and deep-strong coupling regimes, where coupling strengths are comparable to or larger than subsystem energies. Demonstrating dynamics remains an outstanding challenge, the few experiments reaching these regimes being limited to spectroscopy. Here, we employ a circuit quantum electrodynamics chip with moderate coupling between a resonator and transmon qubit to realise accurate digital quantum simulation of deep-strong coupling dynamics. We advance the state of the art in solid-state digital quantum simulation by using up to 90 second-order Trotter steps and probing both subsystems in a combined Hilbert space dimension of ∼80, demonstrating characteristic Schrödinger-cat-like entanglement and large photon build-up. Our approach will enable exploration of extreme coupling regimes and quantum phase transitions, and demonstrates a clear first step towards larger complexities such as in the Dicke model.

## Abstract (web summary)

Realising deep-strong coupling phenomena for interacting light-matter systems remains an experimental challenge. Here, Langford et al. employ a circuit quantum electrodynamics chip with moderate coupling between a resonator and transmon qubit to realise digital quantum simulation of deep-strong coupling dynamics.

## Introduction

Targetted digital quantum simulations^1^ are likely to provide the first demonstrations of quantum advantage for small-scale quantum computers, with applications in fields such as quantum chemistry^2, 3^ and condensed-matter physics^4–7^. In a digital quantum simulator, sequences of discrete interaction components synthesise the evolution of an artificial Hamiltonian, allowing access to more exotic dynamics than the simulator can realise naturally. Systems involving ultrastrong light-matter interactions raise significant challenges for both theoretical analysis^8–13^ and experimental study^14^, making them ripe candidates for exploration via quantum simulation.

Ultrastrong coupling (USC)^9^ of light and matter has been achieved in a range of physical systems, including circuit quantum electrodynamics (cQED)^15–17^, semiconductor quantum well systems^18^, terahertz electron cyclotron transitions^19–21^ and photochromic molecules^22^. Some experiments have demonstrated spectroscopic signatures deep into USC^16, 17, 20^ where the coupling-to-frequency ratio *g*/*ω* ≳ 1 (at so-called deep-strong coupling, or DSC^23^), but a dynamical signature has only been measured at $g{\rm{/}}\omega \sim 0.09$ ^21^.

The standard quantum Rabi model (QRM)^8^ describes the coupling of a two-level atom (energy $\hbar \omega _{\rm{q}}^{\rm{R}}$) to a quantum harmonic field mode (energy $\hbar \omega _{\rm{r}}^{\rm{R}}$) by a field–dipole interaction (energy *ħg* ^R^):

1 $$\frac{{{H_{\rm{R}}}}}{\hbar } = - \frac{{\omega _{\rm{q}}^{\rm{R}}}}{2}{\sigma _{\rm{z}}} + \omega _{\rm{r}}^{\rm{R}}{a^{\dagger} }a + {g^{\rm{R}}}\left( {a + {a^{\dagger} }} \right)\left( {{\sigma ^ + } + {\sigma ^ - }} \right),$$

where $a = \sqrt n \left| {n \!\!- \!\!1} \right\rangle \left\langle n \right|$ and ${\sigma ^ - } = \left| g \right\rangle \left\langle e \right|$ are annihilation operators for field mode and atom, respectively (with creation operators *a* ^†^ and *σ* ^+^), and ${\sigma _{\rm{z}}} = \left| g \right\rangle \left\langle g \right| \!\!-\!\! \left| e \right\rangle \left\langle e \right|$ is the Pauli z-basis operator. Under small coupling $( {{g^{\rm{R}}} \ll \omega _{\rm{q}}^{\rm{R}},\omega _{\rm{r}}^{\rm{R}}} )$, this reduces to the Jaynes–Cummings (JC) model via the rotating-wave approximation:

2 $$\frac{{{H_{{\rm{JC}}}}}}{\hbar } = - \frac{{{\omega _{\rm{q}}}}}{2}{\sigma _{\rm{z}}} + {\omega _{\rm{r}}}{a^\dagger }a + g\left( {a{\sigma ^ + } + {a^\dagger }{\sigma ^ - }} \right),$$

which contains only the excitation-number-conserving interaction terms, *aσ* ^+^ and *a* ^†^ *σ* ^−^, and has an exact solution. In the USC regime $( {{g^{\rm{R}}}\sim \omega _{\rm{q}}^{\rm{R}},\omega _{\rm{r}}^{\rm{R}}} )$, however, the excitation-nonconserving terms *aσ* ^−^ and *a* ^†^ *σ* ^+^ cannot be neglected and only total parity $[ {{\sigma _{\rm{z}}}\mathop {\sum}\nolimits_n {{( { - 1})}^n}\left| n \right\rangle \left\langle n \right|} ]$ is conserved^23^. Without the strong symmetry of number conservation, the full QRM becomes difficult to solve^10^, predicting phenomena such as ground-state entanglement and large ground-state photon populations, which have not yet been observed experimentally. Theory suggests that simulations of the QRM could explore widely varied coupling regimes in architectures like cQED^24–26^, cold atoms^27^ and trapped ions^28^. Simulated QRM dynamics have been observed in restricted regimes in trapped ions, including the Dirac equation ($\omega _{\rm{r}}^{\rm{R}} = 0$, $\omega _{\rm{q}}^{\rm{R}} \ne 0$)^29, 30^ and coupling only ($\omega _{\rm{r}}^{\rm{R}} = 0$, $\omega _{\rm{q}}^{\rm{R}} = 0$)^31, 32^ regimes. A classical analogue simulation of evolution in a restricted subspace of the QRM has been performed in photonic waveguide systems^23, 33^.

Here, we implement an accurate experimental simulation of quantum Rabi model dynamics well into the deep-strong coupling regime using a cQED quantum simulator with only moderate atom–cavity coupling. To achieve this, we implement a digital protocol^24^ with up to 90 second-order Trotter steps. In particular, we significantly extend the protocol by developing a phase-controlled method for tuning the target system parameters that allows us to explore a wide range of relative coupling strengths with high precision. Combining this control with versatile measurements of atom, cavity and joint system properties, we carry out a comprehensive study of quantum Rabi dynamics from ultrastrong to extreme deep-strong coupling. We first investigate the restricted case with zero atomic frequency $( {\omega _{\rm{q}}^{\rm{R}} = 0})$ to demonstrate key signatures verifying the simulation of deep-strong coupling. These include the characteristic collapses and revivals in both atom and cavity parities, coherent oscillations in cavity population reaching large photon numbers, and opposing cavity phase-space trajectories. We then show that the simulated deep-strong coupling leads to conditional nonclassical Schrödinger cat states in the cavity, which verifies the presence of the atom–cavity entanglement arising from coherent deep-strong coupling dynamics. Finally, we study deep-strong coupling dynamics for several nonzero values of atomic frequency (${g^{\rm{R}}}{\rm{/}}\omega _{\rm{q}}^{\rm{R}}$ ≳ 1). This shows that our simulation is able to access the full complexity of the quantum Rabi model, and allows us to develop a heuristic understanding of the expected dynamics in terms of a competition between deep-strong coupling and JC dynamics.

## Results

### Digital quantum Rabi simulator with phase-controlled tuning

**Fig. 1:**
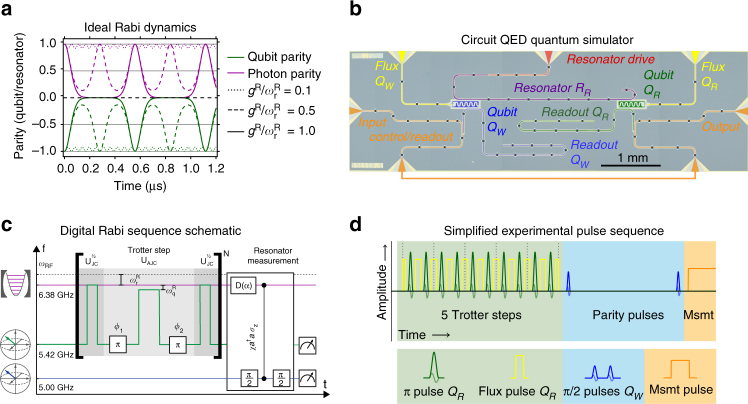
Digital-analogue quantum Rabi simulation using phase-controlled parameter tuning. **a** Parity dynamics of the ideal quantum Rabi model in the degenerate-qubit case $\left( {\omega _{\rm{q}}^{\rm{R}} = 0} \right)$ for qubit (green) and resonator (red) in coupling regimes: $r = {g^{\rm{R}}}{\rm{/}}\omega _{\rm{r}}^{\rm{R}} = 0.1$ (dotted), 0.5 (dashed) and 1.0 (solid). In this example, *g* was chosen to match the experimentally observed value of *g*/2π = 1.79 MHz. **b** Two-transmon, three-resonator cQED chip (detailed description in Supplementary Notes 2 and 3). **c** Sequence schematic for second-order Trotterisation. The rotating frame defining the simulated resonator frequency (*ω*_r_) is controlled via the *Q*_R_ bit-flip pulse phases. **d** Example simplified experimental pulse sequence for 5 Trotter steps followed by a photon parity measurement.

Deep-strong coupling dynamics can produce nontrivial quantum states and significant build-up of photon numbers^23^. Many characteristic dynamical features of DSC can already be seen in the degenerate-qubit limit $\omega _{\rm{q}}^{\rm{R}} = 0$. Here, the interaction-picture Hamiltonian

3 $$\frac{H_{{\rm{R,int}}}}{\hbar} = {\sigma _{\rm{x}}}\left( {{g^{\rm{R}}}{e^{ - i\omega _{\rm{r}}^{\rm{R}}t}}a + {g^{\rm{R}}}{e^{i\omega _{\rm{r}}^{\rm{R}}t}}{a^{\dagger} }} \right)$$

is a coherent drive on the oscillator mode, with an amplitude $\pm {g^{\rm{R}}}{e^{i\omega _{\rm{r}}^{\rm{R}}t}}$ conditioned on the *σ*_x_ basis state of the atom (*σ*_x_ = *σ* ^+^ + *σ* ^−^ is the Pauli x-basis operator). The conditional coupling ±*g* ^R^ coherently displaces the field, but in a continuously rotating direction given by ${e^{i\omega _{\rm{r}}^{\rm{R}}t}}$, creating two diametrically opposite circular trajectories in phase space (see Supplementary Movie 1, with the final frame showing ideal phase-space trajectories, or see later figure on phase-space dynamics). Relating the diameter and circumference of these trajectories, $\pi {\alpha _{{\rm{max}}}} = \dot \alpha {T^{\rm{R}}}$, with the field displacement rate $\dot \alpha = {g^{\rm{R}}}$ and period ${T^{\rm{R}}} = 2\pi {\rm{/}}\omega _{\rm{r}}^{\rm{R}}$, gives a maximum amplitude *α*_max_ = 2*r* set by the relative coupling ratio $r \equiv {g^{\rm{R}}}{{/}}\omega _{\rm{r}}^{\rm{R}}$. Figure 1a illustrates the atomic and photonic parity dynamics (*σ*_z_ and $\mathop {\sum}\nolimits_n {\left( { - 1} \right)^n}\left| n \right\rangle \left\langle n \right|$, respectively) for characteristic coupling regimes, starting in an eigenstate of the uncoupled system, ${\left| e \right\rangle _{\rm{q}}} \otimes {\left| 0 \right\rangle _{\rm{r}}}$. Because this is a superposition of the *σ*_x_ eigenstates ${\left| \pm \right\rangle _{\rm{q}}}\sim {\left| g \right\rangle _{\rm{q}}} \pm {\left| e \right\rangle _{\rm{q}}}$, evolution gives rise to an atom–field entangled state (Bell-cat state)^34^, ${\left| { + , + \alpha } \right\rangle _{{\rm{q,r}}}} - {\left| { - , - \alpha } \right\rangle _{{\rm{q,r}}}}$. For $r \ll 1$, the two trajectories remain virtually indistinguishable, giving evolution closely approximating simple JC dynamics with an atom-field detuning equal to $\omega _{\rm{r}}^{\rm{R}}$ (*cf*. Supplementary Note 7). As *r* increases, the curves start distorting from the sinusoidal JC exchange oscillations (USC regime), until reaching DSC (*r* ≳ 1), where the parities exhibit a characteristic Gaussian-shaped “collapse”, followed by flat plateaus and periodic revivals at multiples of *T* ^R^. The cross-over between these dynamical regimes is related to the maximum distinguishability of the two coherent states of the field. When the paths separate completely, the qubit appears to be in a mixed state, with parity 0.5.

Our circuit QED Rabi simulator uses a hybrid digital-analogue encoding of the atom and field mode, respectively, in a transmon qubit (*Q*_R_)^35^ and a coplanar waveguide resonator (*R*_R_) (energies *ħω*_q_ and *ħω*_r_) (device shown in Fig. 1b). Because the transmon is only weakly anharmonic $( {\omega _{\rm{q}}^{{\rm{0}} - {\rm{1}}} - \omega _{\rm{q}}^{{\rm{1}} - {\rm{2}}} \ll \omega _{\rm{q}}^{{\rm{0}} - {\rm{1}}}} )$, directly increasing the qubit resonator coupling *g* leads to a breakdown in its qubit behaviour at small *r*, and full circuit quantisation shows that DSC cannot be reached for any circuit parameters^36^. Instead, building on the proposal in ref. ^24^, we perform a digital simulation of the QRM for arbitrarily large *r* using a coupling in the manifestly non-USC regime (*r* < 10^−3^). The full Rabi Hamiltonian can be decomposed into two JC-like interactions^24^:

$${H_{\rm{R}}}\left( {{g^{\rm{R}}},\omega _{\rm{r}}^{\rm{R}},\omega _{\rm{q}}^{\rm{R}}} \right) = {H_{{\rm{JC}}}}\left( {g,{\Delta _{\rm{r}}},\Delta _{\rm{q}}^{{\rm{JC}}}} \right) + {H_{{\rm{AJC}}}}\left( {g,{\Delta _{\rm{r}}},\Delta _{\rm{q}}^{{\rm{AJC}}}} \right),$$

where *H*_AJC_ = *σ*_x_ *H*_JC_ *σ*_x_ contains only counter-rotating interaction terms, and the effective Rabi parameters *g* ^R^ = *g*, $\omega _{\rm{r}}^{\rm{R}} = 2{\Delta _{\rm{r}}}$ and $\omega _{\rm{q}}^{\rm{R}} = {\Delta _{\rm{q}}} \equiv \Delta _{\rm{q}}^{{\rm{JC}}} - \Delta _{\rm{q}}^{{\rm{AJC}}}$ are not related to the natural circuit frequencies, but defined relative to a nearby rotating frame (Δ *=* *ω* *−* *ω*_RF_), and can be arbitrarily small. Using the standard method of Trotterization^1^, Rabi dynamics can therefore be simulated into the DSC regime by decreasing Δ_r_ and Δ_q_. Figure 1c illustrates the second-order Trotter step used here (see Methods section and Supplementary Note 9). An asymmetric transmon with two flux-insensitive “sweet” spots^35^ is driven and measured at its lower sweet spot (5.452 GHz) far below the resonator (6.381 GHz), with digital *π* pulses being interleaved with short analogue JC interaction blocks applied by fast frequency-tuning flux pulses^37^. (See Supplementary Note 3 and Supplementary Table 1 for details of the experimental scheme and Supplementary Note 4 for details of how the flux-pulse distortion compensations are calibrated.) Experimentally, a rotating frame is usually defined by the frequency of a drive tone. Here, the choice of rotating frame specifies the required rotation axis of the *π* pulses which create the AJC interaction. By appropriately updating the pulse phases, which are controlled with high precision, we can therefore arbitrarily select the rotating frame detuning from the resonator, even though these pulses are applied far from both resonator and rotating frame (see Methods section).

Numerical modelling of the digital Rabi protocol highlighted several challenges for device design and fabrication (Supplementary Note 2). Most significantly, due to practical flux-pulsing bandwidths which limit the shortest achievable Trotter step, it is challenging to digitise fast compared with the dynamics. Therefore, reaching acceptably low Trotter error in interesting regimes of *r* required small qubit-resonator coupling (here, *g*/2*π* = 1.95 MHz). This also placed constraints on other device parameters, including coherence (for long simulation times), flux-tuning precision and qubit-resonator frequency targetting (due to a very narrow resonance). An extra qubit *Q*_W_ was strongly and dispersively coupled to *R*_R_ to probe the intraresonator quantum state via its photon-dependent frequency shift (−1.26 MHz per photon) using pulse sequences based on Ramsey interferometry. We used *Q*_W_ to implement a range of photon measurements: average photon number with a controllable dynamic range (number meter), average photon parity^38, 39^ (parity meter) and, combining parity measurements with coherent field displacements through an external input coupler, direct Wigner tomography of the resonator. (Full details of the operating principles and calibrations of these different photon measurements are provided in Supplementary Notes 5 and 6.) Qubits were driven and measured through dedicated read-out resonators. A full description of the experimental setup is provided in Supplementary Note 1.

### Comparing qubit and resonator parity dynamics

**Fig. 2:**
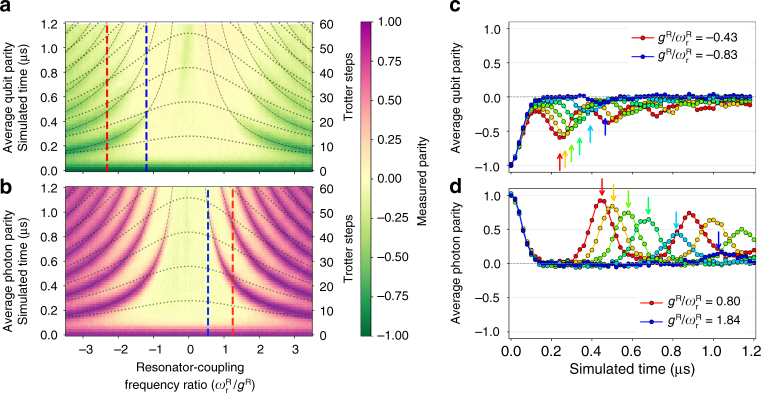
Qubit and resonator parity dynamics of the quantum Rabi model in the degenerate-qubit case. Measured dynamical landscapes for **a** qubit and **b** photon parity dynamics for a broad range of parameters up to 60 Trotter steps, with the extreme DSC regime in the centre decreasing to weaker USC near the edges. The data show clear Gaussian-shaped collapses for all *r*, along with the characteristic plateaus of DSC. Qubit revivals are observed up to *r* ∼ 0.8, while photon parity shows clear revivals up to *r* ∼ 1.8. Vertical line cuts for **c** qubit and **d** photon parity are plotted for evenly spaced $\omega _{\rm{r}}^{\rm{R}}{\rm{/}}{g^{\rm{R}}}$ between the red and blue dashed lines in **a**, **b**, respectively. For *r* ≳ 1.5, some deviation from the expected revival time in the photon parity results from a small residual Kerr-type nonlinearity in the resonator (see also Supplementary Fig. 9) and is correlated with significant photon populations. Arrows in **c**, **d** show expected revival times for each slice. In this and following figures, coupling ratios were calculated using the observed simulated coupling of *g*/2π = 1.79 MHz.

We first experimentally simulate the QRM for the degenerate-qubit case over a wide range of *r*, covering the USC and DSC regimes from *r* ~ 0.3 to *r*→∞ (Fig. 2). We use 60 Trotter steps to simulate 1.2 μs of dynamics (*gt* = 4.68*π*) and measure either qubit or photon parity after each step. (Simulations start in the state ${\left| 1 \right\rangle _{\rm{q}}} \otimes {\left| 0 \right\rangle _{\rm{r}}}$ for all results in the main text, but Supplementary Note 8 shows that the features of DSC dynamics are observed also for ${\left| 0 \right\rangle _{\rm{q}}} \otimes {\left| 0 \right\rangle _{\rm{r}}}$.) A simplified pulse sequence is illustrated in Fig. 1d. The qubit and photon parity dynamics (Fig. 2a, b) show very similar qualitative behaviour, consistent with parity conservation. At all large couplings, the measurements exhibit the Gaussian-shaped parity collapse (set by the simulated *g* ^R^) and flat plateau which are a key signature of DSC dynamics. Fitting the initial qubit data points, we calculate an average *g* ^R^ ≈ 2*π* × 1.79 MHz, slightly lower than the expected *g* ^R^ = *g* ≈ 2*π* × 1.95 MHz determined from independent spectroscopy and vacuum Rabi oscillations. This is consistent with a small residual flux pulse distortion and provides the best estimate for the simulated *g* ^R^ achieved in these experiments. The revival periods *T* ^R^ are in excellent agreement with the predictions of USC Rabi dynamics (dashed curves), and strikingly different from those predicted for a pure JC interaction with the equivalent qubit-resonator detuning $( {{T^{{\rm{JC}}}} = 2\pi\,{\rm{/}}\sqrt {4{g^2} + \Delta _{{\rm{q}} - {\rm{r}}}^2} } )$ (dotted curves) (Supplementary Note 7).

From the observation of parity revivals, combined with the simulated *g* ^R^, we can estimate the range of *r* reached in these simulations. For *g* ^R^/2*π* = 1.79 MHz and *r* = 1 (archetypal DSC), the expected revival time is 0.56 *μ*s. Line cuts for the qubit parity dynamics (Fig. 2c) show revivals beyond 0.4 μs (*r* ~ 0.7). Photon parity revivals, however, persist beyond 1.0 μs (*r* ~ 1.8) (Fig. 2d). This difference again results from photon decay, as confirmed by excellent agreement with numerical modelling which includes cavity decay but no other decoherence (not shown). Photon decay becomes increasingly critical at larger couplings, because even a single decay destroys the qubit-resonator entanglement, and losing a photon becomes increasingly likely for larger photon numbers. The qubit parity revivals rely on entanglement being maintained. This is supported by measurements of reduced qubit entropy, which show that the qubit state collapses to the mixed state, before displaying a revival in purity (Supplementary Note 11). The resonator parity dynamics, however, are more robust to decay and provide a more direct measure of DSC dynamics. Photon parity collapses and revivals prove the field undergoes large-amplitude excursions through phase space even during a single cycle of the resonator period. The difference between qubit and photon parity dynamics is a quantitative signature of breakdown in parity conservation, caused by resonator decay.

### Resonator photon number dynamics

**Fig. 3:**
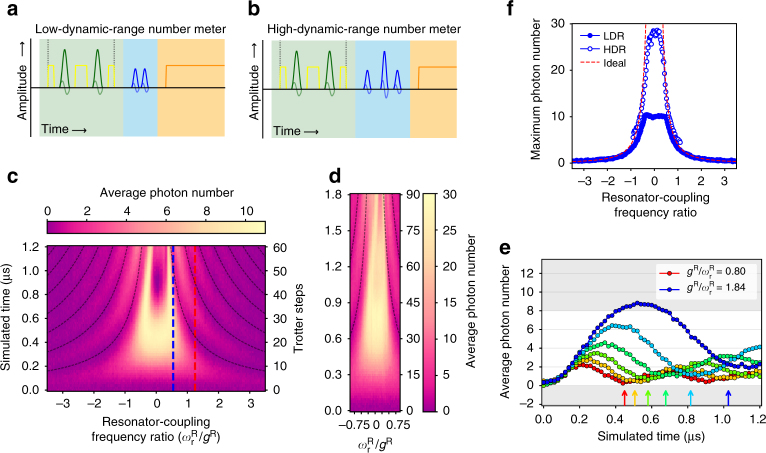
Photon number dynamics of the quantum Rabi model in the degenerate-qubit case. **a**, **b** Average photon number is probed by applying Ramsey and echo-like pulses to *Q*_W_. The effective Ramsey pulse separation *τ* determines the photon dynamic range. Because of finite pulse widths, reaching the small *τ* needed for high dynamic ranges **b** requires an unbalanced ‘echo’-like sequence. **c**, **e** Measured photon number dynamics up to 60 Trotter steps using a low-dynamic-range (LDR) photon number meter ($\tau \sim 18.7$ ns) with a linear range of ∼0–8 photons (indicated by grey regions in **e**). Large photon populations in the resonator highlight the non-conservation of excitation number in the quantum Rabi model. The resonator displays clear oscillations up to *r* > 1.8 in good agreement with the expected qubit revival times (dashed curves). The red feature in the middle reflects the upper limit on the number meter’s dynamic range set by *Q*_W_ ‘population wrapping’ at high photon numbers. **d** Measured photon dynamics up to 90 Trotter steps using a high-dynamic-range (HDR) number meter with $\tau \sim 6.5$ ns and a linear range of ∼0–20 photons, allowing observation of photon oscillations beyond 1.5 μs of simulated time (more than 75 Trotter steps). This data shows the effect of a residual Kerr nonlinearity at high values of *r*. **e** Line slices are plotted for evenly spaced resonator-coupling frequency ratios between the red and blue dashed lines shown in **c**. Grey regions delineate the linear range of the number meter. **f** Maximum measured average photon number for each value of *r* for both LDR and HDR number meters.

We next directly explore the build-up of large photon populations (Fig. 3), another feature of DSC dynamics that contrasts strikingly with the excitation-conserving dynamics expected under weak coupling. Using a Ramsey pulse sequence with small separation *τ*, the excitation probability in *Q*_W_ becomes a measure of average photon number in the resonator (Supplementary Note 5). The dynamic range and sensitivity of this number meter are controlled via *τ* (Fig. 3a, b). Measured with a linear range of ~ 0–8 photons (Fig. 3c), the resonator displays the complementary build-up of photons which causes the collapse of qubit and photon parity, clearly demonstrating the violation of number conservation expected for the QRM. As with photon parity, clear oscillations can be seen out to *r* ~ 1.8 (Fig. 3e). The large central feature appears to deviate from the expected trend, but is in fact due to photon number exceeding the dynamic range of the number meter. To explore this region further, we extended the linear range to ~ 0–20 photons using a number meter with a non-centred refocussing pulse (Fig. 3d) and simulated up to 90 Trotter steps (*gt* = 7.0*π*), allowing photon oscillations beyond 1.5 *μ*s to be observed. This range operated at the limits of approximately uniform driving given the bandwidth of the 12 ns (4*σ*) *Q*_W_ pulses. At *r* ≳ 2, the photon dynamics in Fig. 3c, d are clearly skewed, causing the observed oscillations to deviate from the expected revival period *T* ^R^ (also observable in the photon parity (Fig. 2b). This results from a residual Kerr nonlinearity in *R*_R_ inherited from the dispersively coupled ancilla qubit^40^.

Exploring the resonator oscillations more quantitatively, the maximum photon number in each vertical (constant-*r*) slice (Fig. 3f) compares well with the expected ideal behaviour. The discrepancy between the two curves in the overlapping region results from bandwidth limitations in the high-dynamic-range (HDR) number meter and from limits in linearity of the number-to-probability mapping for *Q*_W_. Because of the sinusoidal conversion, the calibrated value at either end of the range compressed slightly towards the centre from the real photon number. The measurement saturates at the highest *r* even for the HDR meter, suggesting that we observe more than 30 photons (average) building up in the resonator for the strongest DSC regions. Given the Poissonian statistics expected for coherent states, this accesses a resonator subspace of dimension ~ 40 (i.e., a subspace larger than that of 5 qubits). This ability to access large Hilbert spaces with a simple system is an advantage of the analogue resonator encoding.

### Resonator phase-space dynamics

**Fig. 4:**
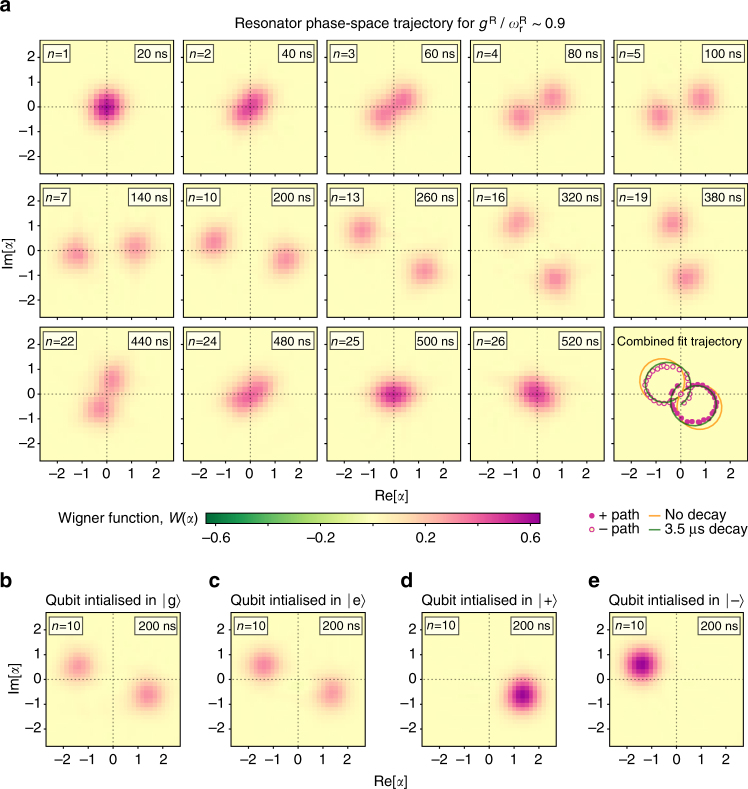
Photon dynamics in phase space in the DSC regime (degenerate-qubit case) from maximum-likelihood Wigner tomography. **a** Selected frames from a ‘movie’ (measured over ∼40 h) showing the phase-space evolution of the resonator reduced state for *r* ∼ 0.9 (frames labelled by Trotter step *n* and simulated time), with the final panel showing the full trajectories determined from 2D double-Gaussian fits to the raw data (the full movie is provided in the Supplementary Movie 1). Plotted tomograms are maximum-likelihood reconstructions of direct Wigner tomography measured data with a systematic phase correction (see Methods section). When the effective drive on the intracavity field created by the Rabi interaction has a strength comparable to the resonator’s natural frequency (i.e., ${g^{\rm{R}}}\sim \omega _{\rm{r}}^{\rm{R}}$), this drive is able to create a significant displacement of the cavity field before the phase-space rotation caused by $\omega _{\rm{r}}^{\rm{R}}$ brings the field back towards the origin. This effect is observed clearly here in the creation of two well-resolved, rotating peaks and subsequent re-coalescence which are characteristic signatures of DSC dynamics. Deviation from the ideal circular trajectories (orange curves) arises from photon decay. The measured trajectory shows excellent agreement with a numerical Trotter simulation at *g* ^R^/2π = 1.79 MHz which includes resonator *T*_1,r_ = 3.5 μs (green curves). From the fits, we calculate an estimated Wigner function width *σ* = 0.526 ± 0.003, instead of the predicted 0.5, indicating a displacement calibration error of ∼5% (Supplementary Note 6). Background noise arises from phase instability of microwave sources and frequency stability of the Wigner qubit over the long measurement. **b**–**e** Conditional phase-space evolution illustrated by the resonator Wigner function for different initial states of *Q*_R_: **b** $\left| 0 \right\rangle$, **c** $\left| 1 \right\rangle$, **d** $\left| + \right\rangle$ and **e** $\left| - \right\rangle$. The phase-space trajectory of *R*_R_ depends on the qubit state in the *σ*_x_ basis, consistent with creation of Bell-cat hybrid entanglement between *Q*_R_ and *R*_R_ of the form: ${\left| + \right\rangle _{\rm{Q}}}{\left| { + \alpha } \right\rangle _{\rm{R}}} - {\left| - \right\rangle _{\rm{Q}}}{\left| { - \alpha } \right\rangle _{\rm{R}}}$.

Combining the parity measurement with coherent displacements from an external drive allows observation of resonator phase-space dynamics using direct Wigner tomography^38, 39^. Figure 4a shows unconditional maximum-likelihood tomograms (ignoring the state of *Q*_R_; see Methods section) measured after each Trotter step with *r* ~ 0.9 (full movie available in Supplementary Movie 1), with the full trajectory obtained from two-dimensional double-Gaussian fits of the raw data. The resonator state displays the clear signatures of DSC dynamics, first separating into two distinct Gaussian (coherent state) peaks which follow opposite circular trajectories before re-coalescing at the origin. The peaks do not return perfectly to the origin because of photon decay, in agreement with a numerical simulation at *g* ^R^/2*π* = 1.79 which includes *T*_1,*r*_ = 3.5 μs (green curves).

### Demonstrating qubit-resonator entanglement

**Fig. 5:**
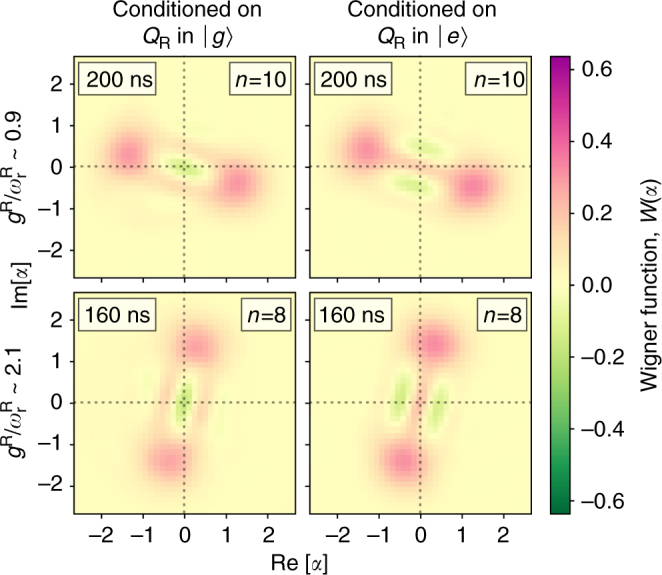
Nonclassical resonator cat states from conditioned DSC-driven entanglement (degenerate-qubit case). The plots show Wigner functions of nonclassical Schrödinger cat states in the Rabi resonator, reconstructed from maximum-likelihood state tomography for two different DSC coupling strengths with ${g^{\rm{R}}}{\rm{/}}\omega _{\rm{r}}^{\rm{R}}\sim 0.9$ (top, *n* = 10 Trotter steps) and ${g^{\rm{R}}}{\rm{/}}\omega _{\rm{r}}^{\rm{R}}\sim 2.1$ (bottom, *n* = 8 Trotter steps), conditioned on measuring *Q*_R_ in $\left| 0 \right\rangle$ (left) and $\left| 1 \right\rangle$ (right). The regions of negativity and visibility of several fringes between the well-resolved coherent state peaks are clear signatures of nonclassicality in the Rabi field mode and demonstrates the coherence and entanglement of the underlying qubit-resonator state. Combined with the qubit conditioning shown in Fig. 4, observing clear cat states for both outcomes of the *Q*_R_ measurement is a clear signature of coherent DSC dynamics.

By capturing the complete resonator quantum state, the Wigner function also enables the demonstration of coherence in DSC dynamics, by contrast with photon parity and number measurements, which are largely insensitive to coherence. Observing this requires correlating the resonator and qubit states, because the coherence is stored in entanglement. We did this in two ways. First, we measured the Wigner function after 10 Trotter steps for *r* ~ 0.9 with *Q*_R_ initialised in states $\left| g \right\rangle$, $\left| e \right\rangle$, $\left| + \right\rangle$ and $\left| - \right\rangle$ (Fig. 4b–e). This showed that the resonator and qubit were correlated, consistent with the expected Bell-cat entanglement. Second, we ran the simulation for *r* ~ 0.9 and 2.1 (8 Trotter steps) with the qubit prepared in the excited state, conditioning the *Q*_W_ measurement on the state of *Q*_R_ in the *σ*_z_ basis (Fig. 5). For the expected Bell-cat state, an outcome of $\left| {g\left( e \right)} \right\rangle$ for *Q*_R_ leaves the resonator in an odd (even) Schrödinger cat state $\left( {\left| \alpha \right\rangle \mp \left| { - \alpha } \right\rangle } \right)$. Numerical modelling shows that only in the DSC regime is negativity in the Wigner function observed for both *Q*_R_ measurement outcomes. The negative regions observed in all the Wigner functions demonstrate nonclassicality for all resonator cat states, which arises from coherence in the underlying Bell-cat entanglement. Reduced visibility is again caused primarily by photon decay, but also by single-shot read-out infidelity (here, ~85–90%) and experimental drift over the long measurements. These different measurements provide clear evidence of qubit-resonator entanglement arising from coherent DSC dynamics.

### Quantum Rabi dynamics in the nondegenerate-qubit case

**Fig. 6:**
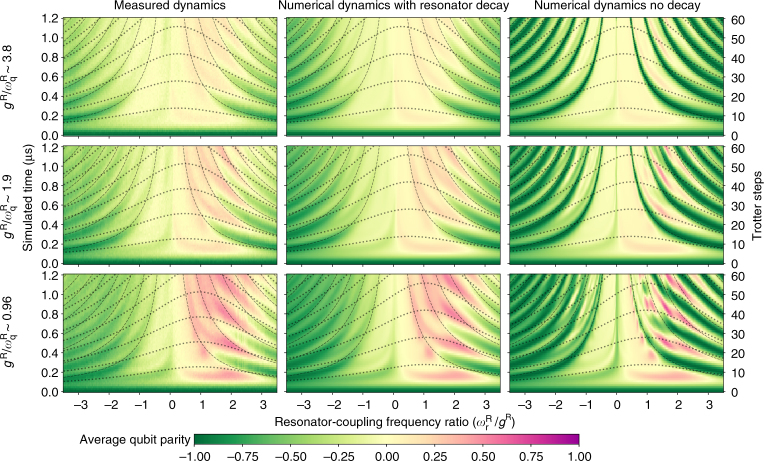
Measured and numerical quantum Rabi model qubit dynamics for nondegenerate qubit frequency. The cases implemented are ${g^{\rm{R}}}{\rm{/}}\omega _{\rm{q}}^{\rm{R}}\sim 4$ (top), ∼2 (middle) and ∼1 (bottom), with the plots showing measured qubit dynamics (left), numerically simulated dynamics of a Trotterised QRM with the measured ${T_{{\rm{1,r}}}}\sim 3.5$ μs included (centre), and ideal Rabi dynamics (right). The results illustrate that the nondegenerate-qubit dynamics do not deviate significantly from the degenerate-qubit case in the regime where $\omega _{\rm{r}}^{\rm{R}} \gg \omega _{\rm{q}}^{\rm{R}}$. The measured dynamics exhibit many qualitative features in good agreement with the ideal QRM and show excellent agreement with the numerical Trotter simulation with decay, indicating that the fidelity of the measured results to the ideal case is limited primarily by resonator decay.

Finally, by detuning the qubit frequency during the AJC half of the Trotter steps [Fig. 1c], we also experimentally simulate dynamics for the nondegenerate-qubit case of the QRM for effective qubit frequencies ${g^{\rm{R}}}{\rm{/}}\omega _{\rm{q}}^{\rm{R}}$ ~ 4, 2 and 1 (Fig. 6). Deviation from the degenerate-qubit case occurs primarily when $\omega _{\rm{r}}^{\rm{R}}$ ≲ $\omega _{\rm{q}}^{\rm{R}}$ ^41^ and these regimes access the full complexity of QRM dynamics. To develop a rough intuition for the expected dynamics, we overlay the plotted landscapes with the expected revival times for both pure degenerate-qubit QRM dynamics and pure nondegenerate-qubit JC (exchange) dynamics (centred around the effective qubit frequency). This illustrates that the ideal dynamics (no decay) (Fig. 6 (right)) can be thought of as a competition between the two cases. As qubit frequency increases, standard JC dynamics begin to emerge, with qubit population oscillations (and increasingly pronounced positive-parity regions) appearing in the collapse-revival dynamics characteristic of the DSC regime. This interpretation and trend become clearer for qubit frequencies $\omega _{\rm{q}}^{\rm{R}}$ larger than the coupling *g* ^R^, where the standard JC exchange dynamics start to dominate (numerical modelling shown for ${g^{\rm{R}}}{\rm{/}}\omega _{\rm{q}}^{\rm{R}}\sim 0.48$ in Supplementary Fig. 12). The measured dynamics (Fig. 6 (left)) capture many features of the ideal case (Fig. 6 (right)), even up to $r \gg 1$. Numerical modelling of the digital QRM simulation including the measured *T*_1,r_ (Fig. 6 (centre)) confirms that simulation fidelity is primarily limited by resonator decay.

## Discussion

Demonstrating stabilisation by decreasing step sizes will be an important part of validating the behaviour of future complex digital simulators achieving quantum advantage^42^. In Supplementary Notes 9 and 10, we showed that using second-order Trotterisation and decreasing the Trotter step size both significantly improved performance. This indicates that the simulation is not limited by an error-per-gate noise floor as in previous cQED simulations^7^, and enables us to linearly increase the number of Trotter steps for increasing simulated time, rather than keeping the number fixed^3, 6, 7^. This is an important step towards the quadratic scaling needed for universal quantum simulation^1^. In combination, these achievements advance solid-state quantum simulators based on cQED to a digital performance previously attained only in trapped-ion systems^5^.

Interestingly, a QRM simulator even has some direct advantages over natural USC systems. Although large couplings can lead to ground-state entanglement and significant ground-state photon populations, these potentially interesting ground states are not readily accessible in natural USC systems^14, 36, 43^ without the ability to rapidly (nonadiabatically) tune or switch off the ultrastrong coupling. In systems where the coupling reaches many gigahertz, tuning system parameters on this timescale represents a significant technical challenge^16, 17^. In our simulator, however, cavity photons are always real (not virtual), detectable and usable, and it is straightforward to nonadiabatically tune system parameters to implement quantum quenches^44^. This makes a cQED chip with natural JC interactions an ideal platform to explore the preparation of interesting ground states in future experiments. The challenge is that the simulator decay processes differ from those in a natural USC system and do not move the system towards the USC ground state^11^. This highlights the need to improve *T*_1,r_ so that photon decay does not limit the dynamics. It should be possible to improve *T*_1,r_ 10-fold using novel processing methods^45^. However, an interesting next step will be to determine the effective USC decay resulting from simulator-frame resonator decay.

Finally, the phase technique we have developed to define a rotating frame via single-qubit pulses introduces a precise and flexible paradigm for engineering artificial Hamiltonians which can be applied across architectures such as trapped ions and cold atoms^5, 27, 28^. In combination with the number of Trotter steps demonstrated, the technique will allow accurate simulation of the time-dependent Hamiltonians^5, 7, 46^ required to perform adiabatic preparation of USC ground states. It is therefore ideally suited for exploring novel quantum phase transitions relying on extreme coupling regimes recently identified for the QRM^27, 47, 48^. Furthermore, by extending to small-scale Dicke model systems^24, 26^, it will avoid the problem of additional nonlinear evolution terms^26^ which have been suggested to prevent the onset of a long-predicted superradiant phase transition in a range of physical systems^12, 13, 36, 49^.

## Methods

### Phase-controlled Trotterisation of the quantum Rabi model

In the digital QRM simulation proposed in ref. ^24^, the effective parameters of the simulated Rabi Hamiltonian are *g* ^R^ = *g*, $\omega _{\rm{r}}^{\rm{R}} = 2{\Delta _{\rm{r}}}$ and $\omega _{\rm{q}}^{\rm{R}} = \Delta _{\rm{q}}^{{\rm{JC}}} - \Delta _{\rm{q}}^{{\rm{AJC}}}$, where Δ_r_ = *ω*_r_ − *ω*_RF_ and Δ_q_ = *ω*_q_ − *ω*_RF_ are defined relative to a rotating frame. This rotating frame is essential to reaching DSC with weakly anharmonic transmon qubits, by allowing us to tune the simulated $\omega _{\rm{r}}^{\rm{R}}$ and $\omega _{\rm{q}}^{\rm{R}}$. Typically, the frequency of a rotating frame is set by a physical generator or drive signal that defines a rotation or a measurement basis. In the digital simulation, the rotating frame is still abstract, since no drive is used to induce an interaction. Here we describe a method we have developed for controlling the frequency of the rotating frame which is simple, high-resolution and flexible.

The basic intuition is that the bit flips in ref. ^24^, which convert every second JC interaction into an effective AJC interaction, are the only concrete operations which take place in the otherwise abstract rotating frame. In any Trotter step, the frequency of the rotating frame is therefore defined by the rotation axes of the bit-flip pulses (i.e., the absolute pulse phase), but these flips are driven by microwave pulses at a frequency far (~1 GHz) below the resonator, at the qubit’s bottom sweet spot. Nevertheless, while the drive generator’s phase continuously and rapidly rotates relative to the resonator, the drive pulses can be effectively locked to the resonator frequency by discretely updating the pulse phase at each pulse. This is achieved by advancing the phase of each pulse by an amount proportional to the elapsed time between pulses. An arbitrary offset frequency from the resonator is then straightforwardly achieved by correcting this phase advance by an amount proportional to the Trotter step size. Interestingly, in the scheme of ref. ^24^, because the simulated resonator frequency (but not the qubit frequency) is sensitive to the absolute detuning from the rotating frame, this effective qubit offset frequency tunes the frequency of the resonator (but not the qubit).

We now derive the analytical relation between the bit-flip pulse phases and the rotating frame frequency in the simulation. We start by writing down the full Trotter step and then derive the effective Hamiltonian implemented by this step given the lowest-order Trotter approximation. The symmetric, second-order Trotter step for the digital QRM simulation is:

4 $$U_{\rm{R}}^{{\rm{Tr}}}\left( \tau \right) = U_{{\rm{JC}}}^{\frac{1}{2}}\left( \tau \right){U_{{\rm{AJC}}}}\left( \tau \right)U_{{\rm{JC}}}^{\frac{1}{2}}\left( \tau \right),$$

where *U*_JC_(*τ*) = exp(−*iH*_JC_ *τ*/*ħ*) and an arbitrary AJC step

5 $${U_{{\rm{AJC}}}}\left( \tau \right) = {R_{{\phi _2}}}\left( \pi \right){\rm{exp}}\left( {\frac{{ - i{H_{{\rm{JC}}}}\tau }}{\hbar }} \right){R_{{\phi _1}}}\left( \pi \right),$$

is defined by the phases used to set the rotation axes *ϕ*_1,2_ of the bit flips *R*_ϕ_(*π*). Writing the JC Hamiltonian in the rotating frame of the resonator, and using the identity *R*_*ϕ*_(*π*) = *R*_z_(*ϕ*)*R*_x_(*π*)*R*_z_(−*ϕ*) = *R*_z_(2*ϕ*)*σ*_x_ = *σ*_x_ *R*_z_(−2*ϕ*), gives:

6 $${U_{{\rm{AJC}}}}\left( \tau \right) = {R_{\rm{z}}}\left( {2{\phi _2}} \right){\sigma _{\rm{x}}}\,{\rm{exp}}\left[ { - i{\Delta _{{\rm{q}} - {\rm{r}}}}\tau {\sigma _{\rm{z}}}{\rm{/}}2 - i\epsilon \left( {a{\sigma ^ + } + {a^\dagger }{\sigma ^ - }} \right)} \right]{\sigma _{\rm{x}}}{R_{\rm{z}}}\left( { - 2{\phi _1}} \right),$$

7 $$= {\rm exp}\left( { - i\Delta \phi {\sigma _{\rm{z}}}{\rm{/}}2} \right){\rm{exp}}\left( { - i{\phi _\Sigma }{\sigma _{\rm{z}}}{\rm{/}}2} \right){\rm{exp}}\left[ {i{\Delta _{{\rm{q}} - {\rm{r}}}}\tau {\sigma _{\rm{z}}}{\rm{/}}2 - i\epsilon \left( {a{\sigma ^ - } + {a^\dagger }{\sigma ^ + }} \right)} \right] \\ {\rm{exp}}\left( {i{\phi _\Sigma }{\sigma _{\rm{z}}}{\rm{/}}2} \right){\rm{exp}}\left( { - i\Delta \phi {\sigma _{\rm{z}}}{\rm{/}}2} \right),$$

8 $$= {\rm{exp}}\left( { - i\Delta \phi {\sigma _{\rm{z}}}{\rm{/}}2} \right){\rm{exp}}\left[ {i{\Delta _{{\rm{q}} - {\rm{r}}}}\tau {\sigma _{\rm{z}}}{\rm{/}}2 - i\epsilon \left( {a{\sigma ^ - }{e^{ - i{\phi _\Sigma }}} + {a^\dagger }{\sigma ^ + }{e^{i{\phi _\Sigma }}}} \right)} \right] \\ {\rm{exp}}\left( { - i\Delta \phi {\sigma _{\rm{z}}}{\rm{/}}2} \right),$$

where $\epsilon$ = *gτ*, *ϕ*_Σ_ = *ϕ*_1_ + *ϕ*_2_, Δ*ϕ* = *ϕ*_2_ − *ϕ*_1_, ${\Delta _{{\rm{q}} - {\rm{r}}}} = \Delta _{{\rm{q}} - {\rm{r}}}^{{\rm{JC}}} - \Delta _{{\rm{q}} - {\rm{r}}}^{{\rm{AJC}}}$, and we have set $\Delta _{{\rm{q}} - {\rm{r}}}^{{\rm{JC}}} = 0$. Equation (8) is reached by noting that ${e^{ - i{\phi _\Sigma }{\sigma _{\rm{z}}}/2}}{\sigma ^ \pm }{e^{i{\phi _\Sigma }{\sigma _{\rm{z}}}/2}} = {\sigma ^ \pm }{e^{ \pm i{\phi _\Sigma }}}$.

Next, noting that $\Delta \phi = \pi \omega _{\rm{r}}^{\rm{R}}\tau \ll 1$ if $\tau \ll 1{\rm{/}}\omega _{\rm{r}}^{\rm{R}}$, and providing the Trotter conditions $\epsilon = g\tau \ll 1$ and ${\Delta _{{\rm{q}} - {\rm{r}}}}\tau \ll 1$ are fulfilled, we can combine exponentials in Eq. (8) using a Trotter approximation to give:

9 $${U_{{\rm{AJC}}}}\left( \tau \right) \approx {\rm{exp}}\left[ { - i\Delta \phi {\sigma _{\rm{z}}} + i{\Delta _{{\rm{q}} - {\rm{r}}}}\tau {\sigma _{\rm{z}}}{\rm{/}}2 - i\epsilon \left( {a{\sigma ^ - }{e^{ - i{\phi _\Sigma }}} + {a^\dagger }{\sigma ^ + }{e^{i{\phi _\Sigma }}}} \right)} \right].$$

Combining the JC and AJC steps with a further Trotter approximation then gives the full Trotter step

10 $$U_{\rm{R}}^{{\rm{Tr}}}\left( \tau \right) \approx {\rm{exp}}\left[ {i\left( { - 2\Delta \phi + {\Delta _{{\rm{q}} - {\rm{r}}}}\tau } \right)\frac{{{\sigma _{\rm{z}}}}}{2} - i\epsilon \left( {a{\sigma ^ + } + {a^\dagger }{\sigma ^ - } + a{\sigma ^ - }{e^{ - i{\phi _\Sigma }}} + {a^\dagger }{\sigma ^ + }{e^{i{\phi _\Sigma }}}} \right)} \right].$$

So far, we have considered arbitrary *ϕ*_1_ and *ϕ*_2_. In the experiment, however, we keep Δ*ϕ* constant for all sequential pairs of bit flips. Specifically, for the *n*th Trotter step, the two phases are *ϕ*_1_ = *ϕ*_0_ + (2*n* − 2)Δ*ϕ* and *ϕ*_2_ = *ϕ*_0_ + (2*n* − 1)Δ*ϕ*, where the choice of *ϕ*_0_ has no effect on the dynamics. Setting *ϕ*_0_ = 3Δ*ϕ*/2 gives *ϕ*_Σ_ = 4*n*Δ*ϕ*, and the *n*th Trotter step can be rewritten in terms of a frequency *ω*_0_ = 2Δ*ϕ*/*τ* and a simulated time *t*_*n*_ = *nτ*:

11 $$U_{\rm{R}}^{\left( n \right)}\left( \tau \right) = {\rm{exp}}\left[ {i\left( { - {\omega _0} + {\Delta _{{\rm{q}} - {\rm{r}}}}} \right)\tau \frac{{{\sigma _{\rm{z}}}}}{2} - i\epsilon \left( {a{\sigma ^ + } + {a^\dagger }{\sigma ^ - } + a{\sigma ^ - }{e^{ - i2{\omega _0}{t_n}}} + {a^\dagger }{\sigma ^ + }{e^{i2{\omega _0}{t_n}}}} \right)} \right].$$

which corresponds to an effective Hamiltonian:

12 $$\frac{{{{\tilde H}_{{\rm{eff}}}}}}{\hbar } = \left( {{\omega _0} - {\Delta _{{\rm{q}} - {\rm{r}}}}} \right)\frac{{{\sigma _{\rm{z}}}}}{2} + g\left( {a{\sigma ^ + } + {a^\dagger }{\sigma ^ - } + a{\sigma ^ - }{e^{ - i2{\omega _0}t}} + {a^\dagger }{\sigma ^ + }{e^{i2{\omega _0}t}}} \right).$$

Until this point, the calculation has been carried out with both qubit and resonator in a frame rotating with the resonator. We now transform ${\tilde H_{{\rm{eff}}}}$ into a rotating frame where both qubit and resonator are rotating at frequency (−*ω*_0_), i.e., with *H*_0_ = −*ħω*_0_(−*σ*_z_/2 + *a* ^†^ *a*), giving a new effective Hamiltonian:

13 $$\frac{{{H_{{\rm{eff}}}}}}{\hbar } = - {\Delta _{{\rm{q}} - {\rm{r}}}}\frac{{{\sigma _{\rm{z}}}}}{2} + {\omega _0}{a^\dagger }a + g\left( {a + {a^\dagger }} \right)\left( {{\sigma ^ + } + {\sigma ^ - }} \right).$$

This completes the mapping of the phase-controlled Trotterisation into the form of a simulated Rabi Hamiltonian and we can now identify the effective simulated parameters *g* ^R^ = *g*, $\omega _{\rm{q}}^{\rm{R}} = {\Delta _{{\rm{q}} - {\rm{r}}}}$ and $\omega _{\rm{r}}^{\rm{R}} = {\omega _0} = 2\Delta \phi {\rm{/}}\tau$. Note that the final frame transformation takes place in the simulated Hilbert space, i.e., with frequency *ω*_0_ defined relative to simulated time. Consequently, the frequency of the abstract rotating frame in ref. ^24^, defined in the laboratory reference frame of the cQED simulator, is less by a factor 2, i.e., *ω*_RF_ = *ω*_0_/2.

Here, we have shown how to engineer a virtual rotating frame by applying virtual phase corrections via updating the rotation axis of subsequent drive pulses^50^ in the stroboscopic context of Trotterised digital quantum simulations. This technique should be broadly applicable in the context of Trotterised quantum simulations, although some details or interpretation may vary depending on the specific simulation. For example, it could be applied virtually unmodified to implement the digital Ising model simulations with interacting spins from ref. ^6^, where phase gates were instead implemented via physical detunings of the qubits (as also done in ref. ^5^). More generally, in Trotterised dynamics, a continuous frequency detuning is to lowest order identical to a discrete phase gate applied in each Trotter step. In any case where a gate is implemented using an exchange-type interaction, frequency detunings can therefore be effectively transferred between different circuit elements and mapped onto the most easily controllable element. This turns the theoretical aide of moving between interaction pictures into a concrete experimental tool. If the Trotter step also includes single-element control pulses, then these can often be modified to also incorporate the phase gate. If this option is not available (e.g., see the digital JC simulation in Supplementary Note 7) then the phase correction can still be implemented directly. In our case, a simulated frequency detuning was applied to a resonator (which was not easily tunable) by virtually applying a discrete phase update to the qubit via the drive phase of the bit-flip pulses.

### Trotter step

For a second-order Trotter step with simulated time *τ*, the Trotter step consists of three flux pulses (*τ*/2, *τ* and *τ*/2) and two single-qubit rotations with buffers separating the different gates. Adjacent *τ*/2 flux pulses from neighbouring Trotter steps are implemented as a single flux pulse of length *τ*. Each flux pulse was followed by a 5 ns phase-compensation flux pulse (Supplementary Note 7). For most of the data presented in this work, the simulated *τ* = 20 ns. The qubit drive pulses on *Q*_R_ were 16 ns total duration (4*σ*) and the pulses buffers were 10 ns. The total Trotter step for *τ* = 20 ns was therefore *τ*_step_ = 122 ns. In addition to the drive-pulse phase advance required to define $\omega _{\rm{r}}^{\rm{R}}$, another linear phase advance $\Delta \phi = \left( {\omega _{\rm{q}}^{{\rm{drive}}} - {\omega _{\rm{r}}}} \right){\tau _{{\rm{step}}}}{\rm{/}}2$ is required to compensate the rapid rotation of the qubit drive with respect to the resonator frequency.

### Qubit control

Qubit rotations were implemented using DRAG pulses^51, 52^, with a Gaussian envelope in the *X* quadrature and a derivative-of-Gaussian envelope in the *Y* quadrature. The 4*σ* pulse durations were 16 ns for *Q*_R_ and 12 ns for *Q*_W_. The performance of the Trotter sequences, which contained up to 180 bit-flip pulses, was very sensitive to details of the *Q*_R_ pulse calibrations. In particular, the drive amplitude was calibrated using a sequence of 50 *π*-pulse pairs preceding a single *π*/2 pulse. All parameters were typically calibrated just before launching a long measurement. The drive amplitude was intermittently recalibrated during the scans. Because only two or three pulses were applied to *Q*_W_ for the photon measurements, it was optimised using the AllXY sequence^53^ of 21 combinations of two *σ*_x_ and *σ*_y_ rotations (either *π*/2 or *π*). The frequency of *Q*_W_ was regularly calibrated during photon measurements using Ramsey sequences.

### Wigner tomography reconstructions

Tomograms shown in Figs. 4 and 5 are maximum-likelihood reconstructions^54, 55^ of the resonator quantum state from direct Wigner tomography measurements^39^. The Wigner function at a phase-space position *α* is:

14 $$W\left( \alpha \right) = \frac{2}{\pi }{\rm{Tr}}\left[ {{\Pi}{D^\dagger }\left( \alpha \right){\rho _{\rm{r}}}D\left( \alpha \right)} \right] = \frac{2}{\pi }{\rm{Tr}}\left[ {{M_\alpha }{\rho _{\rm{r}}}} \right],$$

where *ρ*_r_ is the resonator density matrix, ${\Pi} = \mathop {\sum}\nolimits_n {\left( { - 1} \right)^n}\left| n \right\rangle \left\langle n \right|$ is the photon parity operator and *D*(*α*) is the coherent displacement operator. For each measured *α*, we calculated *M*_*α*_ = *D*(*α*)Π*D* ^†^(*α*) using an operator dimension much larger than the largest ${\left| \alpha \right|^2}$ in the measured phase space, to avoid edge effects when calculating *D*(*α*). The *M*_*α*_ were then truncated to a maximum photon number sufficient to capture all of the reconstructed state, but small enough to allow fast reconstructions and ensure an informationally complete set of operators (*n*_max_ = 12 and 8 for tomograms in Figs. 4 and 5, respectively). The maximum-likelihood reconstruction was carried out using convex optimisation^56, 57^. In Fig. 4, a systematic phase correction was applied to the density matrices to correct for a miscalibration of the resonator drive phase used in the coherent displacement. Finally, the reconstructed density matrix was then used to calculate the plotted Wigner functions.

### Data availability

Data and related analysis are available from the corresponding author on request.

## Electronic supplementary material

Supplementary Information

Description of Additional Supplementary Files

Supplementary Movie 1

## References

[1] Lloyd S (1996). Universal quantum simulators. Science.

[2] Kassal I, Jordan SP, Love PJ, Mohseni M, Aspuru-Guzik A (2008). Polynomial-time quantum algorithm for the simulation of chemical dynamics. Proc. Natl Acad. Sci. USA.

[3] O’Malley PJJ (2016). Scalable quantum simulation of molecular energies. Phys. Rev. X.

[4] Abrams DS, Lloyd S (1997). Simulation of many-body fermi systems on a universal quantum computer. Phys. Rev. Lett..

[5] Lanyon BP (2011). Universal digital quantum simulation with trapped ions. Science.

[6] Salathé Y (2015). Digital quantum simulation of spin models with circuit quantum electrodynamics. Phys. Rev. X.

[7] Barends R (2015). Digital quantum simulation of fermionic models with a superconducting circuit. Nat. Commun..

[8] Rabi I (1936). On the process of space quantization. Phys. Rev..

[9] Ciuti C, Bastard G, Carusotto I (2005). Quantum vacuum properties of the intersubband cavity polariton field. Phys. Rev. B.

[10] Braak D (2011). Integrability of the rabi model. Phys. Rev. Lett..

[11] Beaudoin F, Gambetta JM, Blais A (2011). Dissipation and ultrastrong coupling in circuit qed. Phys. Rev. A.

[12] Nataf P, Ciuti C (2010). No-go theorem for superradiant quantum phase transitions in cavity qed and counter-example in circuit qed. Nat. Commun..

[13] Viehmann O, von Delft J, Marquardt F (2011). Superradiant phase transitions and the standard description of circuit qed. Phys. Rev. Lett..

[14] Lolli J, Baksic A, Nagy D, Manucharyan VE, Ciuti C (2015). Ancillary qubit spectroscopy of vacua in cavity and circuit quantum electrodynamics. Phys. Rev. Lett..

[15] Niemczyk T (2010). Circuit quantum electrodynamics in the ultrastrong-coupling regime. Nat. Phys..

[16] Forn-Daz P (2017). Ultrastrong coupling of a single artificial atom to an electromagnetic continuum in the nonperturbative regime. Nat. Phys..

[17] Yoshihara F (2017). Superconducting qubitoscillator circuit beyond the ultrastrong-coupling regime. Nat. Phys..

[18] Günter G (2009). Sub-cycle switch-on of ultrastrong light-matter interaction. Nature.

[19] Scalari G (2012). Ultrastrong coupling of the cyclotron transition of a 2d electron gas to a thz metamaterial. Science.

[20] Maissen C (2014). Ultrastrong coupling in the near field of complementary split-ring resonators. Phys. Rev. B.

[21] Zhang Q (2016). Collective non-perturbative coupling of 2d electrons with high-quality-factor terahertz cavity photons. Nat. Phys..

[22] Schwartz T, Hutchison JA, Genet C, Ebbesen TW (2011). Reversible switching of ultrastrong light-molecule coupling. Phys. Rev. Lett..

[23] Casanova J, Romero G, Lizuain I, Garca-Ripoll JJ, Solano E (2010). Deep strong coupling regime of the jaynes-cummings model. Phys. Rev. Lett..

[24] Mezzacapo A (2014). Digital quantum rabi and dicke models in superconducting circuits. Sci. Rep..

[25] Ballester D, Romero G, Garca-Ripoll JJ, Deppe F, Solano E (2012). Quantum simulation of the ultrastrong-coupling dynamics in circuit quantum electrodynamics. Phys. Rev. X.

[26] Lamata L (2017). Digital-analog quantum simulation of generalized dicke models with superconducting circuits. Sci. Rep..

[27] Felicetti S (2017). Quantum rabi model in the brillouin zone with ultracold atoms. Phys. Rev. A.

[28] Pedernales J (2015). Quantum rabi model with trapped ions. Sci. Rep..

[29] Gerritsma R (2010). Quantum simulation of the dirac equation. Nature.

[30] Gerritsma R (2011). Quantum simulation of the klein paradox with trapped ions. Phys. Rev. Lett..

[31] Lo HY (2015). Spin-motion entanglement and state diagnosis with squeezed oscillator wavepackets. Nature.

[32] Kienzler D (2016). Observation of quantum interference between separated mechanical oscillator wave packets. Phys. Rev. Lett..

[33] Crespi A, Longhi S, Osellame R (2012). Photonic realization of the quantum rabi model. Phys. Rev. Lett..

[34] Vlastakis B (2015). Characterizing entanglement of an artificial atom and a cavity cat state with Bell’s inequality. Nat. Commun..

[35] Koch J (2007). Charge-insensitive qubit design derived from the Cooper pair box. Phys. Rev. A.

[36] Jaako T, Xiang ZL, Garcia-Ripoll JJ, Rabl P (2016). Ultrastrong-coupling phenomena beyond the dicke model. Phys. Rev. A.

[37] DiCarlo L (2009). Demonstration of two-qubit algorithms with a superconducting quantum processor. Nature.

[38] Bertet P (2002). Direct measurement of the wigner function of a one-photon fock state in a cavity. Phys. Rev. Lett..

[39] Vlastakis B (2013). Deterministically encoding quantum information using 100-photon Schrödinger cat states. Science.

[40] Bourassa J, Beaudoin F, Gambetta JM, Blais A (2012). Josephson-junction-embedded transmission-line resonators: From kerr medium to in-line transmon. Phys. Rev. A.

[41] Albert VV, Scholes GD, Brumer P (2011). Symmetric rotating-wave approximation for the generalized single-mode spin-boson system. Phys. Rev. A.

[42] Cirac JI, Zoller P (2012). Goals and opportunities in quantum simulation. Nat. Phys..

[43] Andersen CK, Blais A (2017). Ultrastrong coupling dynamics with a transmon qubit. N. J. Phys..

[44] Zurek W (1985). Cosmological experiments in superfluid helium?. Nature.

[45] Bruno A (2015). Reducing intrinsic loss in superconducting resonators by surface treatment and deep etching of silicon substrates. Appl. Phys. Lett..

[46] Barends R (2016). Digitized adiabatic quantum computing with a superconducting circuit. Nature.

[47] Hwang MJ, Puebla R, Plenio MB (2015). Quantum phase transition and universal dynamics in the rabi model. Phys. Rev. Lett..

[48] Puebla R, Hwang MJ, Plenio MB (2016). Excited-state quantum phase transition in the rabi model. Phys. Rev. A.

[49] De Liberato S (2014). Light-matter decoupling in the deep strong coupling regime: The breakdown of the purcell effect. Phys. Rev. Lett..

[50] Steffen M, Vandersypen LMK, Chuang IL (2000). Simultaneous soft pulses applied at nearby frequencies. J. Magn. Reson..

[51] Motzoi F, Gambetta JM, Rebentrost P, Wilhelm FK (2009). Simple pulses for elimination of leakage in weakly nonlinear qubits. Phys. Rev. Lett..

[52] Chow JM (2010). Optimized driving of superconducting artificial atoms for improved single-qubit gates. Phys. Rev. A.

[53] Reed, M. D. *PhD Dissertation* (Yale University, New Haven, 2013).

[54] Hradil Z (1997). Quantum-state estimation. Phys. Rev. A.

[55] James DFV, Kwiat PG, Munro WJ, White AG (2001). Measurement of qubits. Phys. Rev. A.

[56] Boyd, S. *Convex Optimization* 1st ed (Cambridge University Press, Cambridge, 2004).

[57] Langford NK (2013). Errors in quantum tomography: diagnosing systematic versus statistical errors. N. J. Phys..

