# Supplementary material for: Experimentally simulating the dynamics of quantum light and matter at deep-strong coupling
Source: Nat Commun. 2017 Nov 23;8:1715. doi: 10.1038/s41467-017-01061-x (PMC5700074; doi:10.1038/s41467-017-01061-x)
Supplement: Supplementary file 1 — Supplementary Information [file 41467_2017_1061_MOESM1_ESM.pdf]

Supplementary Note 1: Experimental setup

The sample and low-temperature microwave components were mounted inside magnetic and infrared radiation shielding consisting of two layers of cryogenic mu metal around a layer of aluminium, with an internal layer of copper foil coated in a mixture of silicon carbide and Stycast (2850 FT) [1]. Microwave coaxial cables are connected to the PCB-mounted chip via non-magnetic SMP connectors (Rosenberger).

The qubit drive and read-out tones are sent through two dedicated feedlines which are connected via a short coaxial cable off-chip. The input line for the qubit drives is filtered at the mixing chamber with 30 dB cold attenuation, a small home-built inline eccosorb filter and a 10 GHz low-pass filter (K&L 6L250-10000/T20000-0/0). [The resonator input line filter is 8 GHz low-pass (K&L 6L250-8000/T18000-0/0).] The output line passes through two 3–12 GHz isolators (Pamtech CWJ1019K) and a circulator (Quinstar CTH0408KCS) mounted above the mixing chamber on the way to a 4–8 GHz cryogenic HEMT amplifier (Low-Noise Factory LNF-LNC4_8A), two room-temperature amplifiers (Miteq AFS3-04000800-10-ULN, then AFS3-00101200-35-ULN-R), RF demodulation (Marki 0618LXP IQ mixer) and amplification, and finally digitised in a data acquisition card (AlazarTech ATS9870). The flux-bias lines are filtered at the mixing chamber with 1.35 GHz low-pass filters (Minicircuits VLFX-1350) followed by home-built eccosorb filters. All input lines are thermalised with 20 dB attenuators mounted at the 4 K plate. The microwave input lines and output line are connected to the fridge through a DC block.

Qubit and resonator drive pulses are created via single-sideband modulation with IQ mixers and generated by two arbitrary waveform generators (AWGs; Tektronix AWG5014). We use a 3–7 GHz IQ mixer (Marki 0307MXP) for the resonator and two custom-built 4–8.5 GHz IQ mixers (QuTech F1c: DC–3.5 GHz IF bandwidth) for the qubit drives. The qubit drive pulses were amplified by a high-power (35 dB) microwave amplifier (Minicircuits ZV-3W-183) before passing through a 5.5 GHz low-pass filter (Minicircuits LFCN 5500+) to minimise amplifier noise at the readout resonator frequencies.

Most microwave units receive a 10 MHz reference from a microwave generator (Agilent E8257D) via a home-built distribution unit. However, the generators used for driving Q_R and R_R (R&S SGS100A) synchronised directly via a 1 GHz reference. This was critical to achieving the phase stability required to measure R_R Wigner functions during measurement runs lasting up to 40 hours. The frequencies for these two generators were also always set to a multiple of the trigger repetition rate (5 kHz), to ensure a stable phase relationship. For phase-sensitive measurements, a 500 MHz scope (Rigol DS4034) monitored the relative trigger timing between the master and slave AWGs to select consistent delay configurations between the AWG outputs.

Home-built low-noise current sources mounted in a TU Delft IVVI-DAC2 rack provided precision DC bias currents for flux tuning of the qubit frequencies. The DC bias for Q_R was combined with the amplified output of one channel of the master AWG (the same as used for generating Q_R drive pulses) using a reactive bias tee (Minicircuits ZFBT-6GW+). The flux pulses from the AWG were amplified using a home-built 2 V/V flux-pulse amplifier.

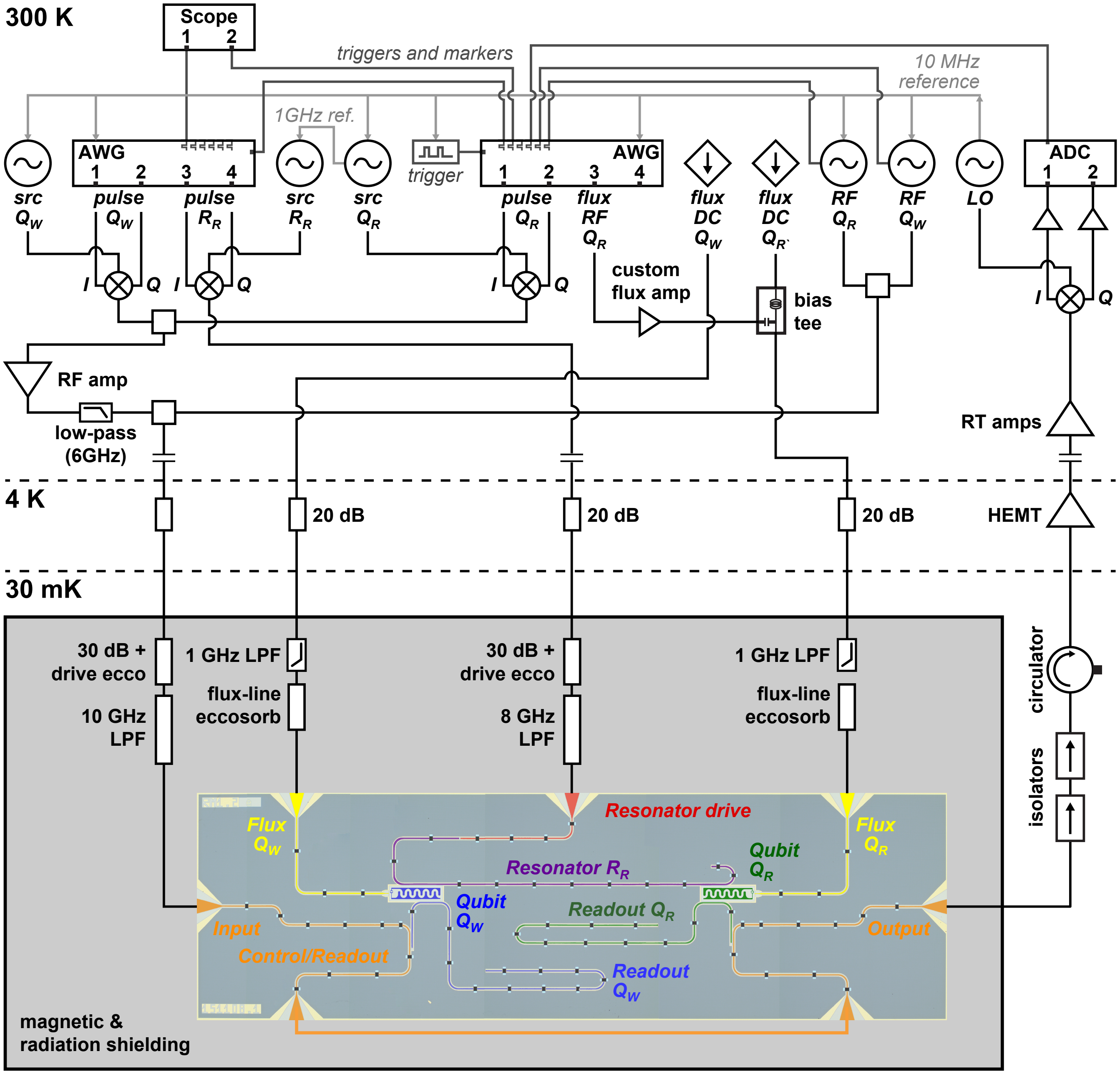

Supplementary Figure 1. Experimental schematic showing the connectivity of microwave electronics and components in and outside the dilution refrigerator. The sample mounted below the mixing chamber typically remained at around 30 mK. Qubit and resonator drive lines and flux-bias lines were thermalised and attenuated at the 4-K and 30-mK stages and were low-pass filtered before arriving at the sample. The qubits and resonator drive pulses were generated by AWGs and IQ mixers. Home-built low-noise current sources provided DC bias currents for qubit frequency tuning, which were combined with fast frequency-tuning bias pulses using reactive bias tees. AWG markers provided the gating for pulse-modulated measurement pulses.

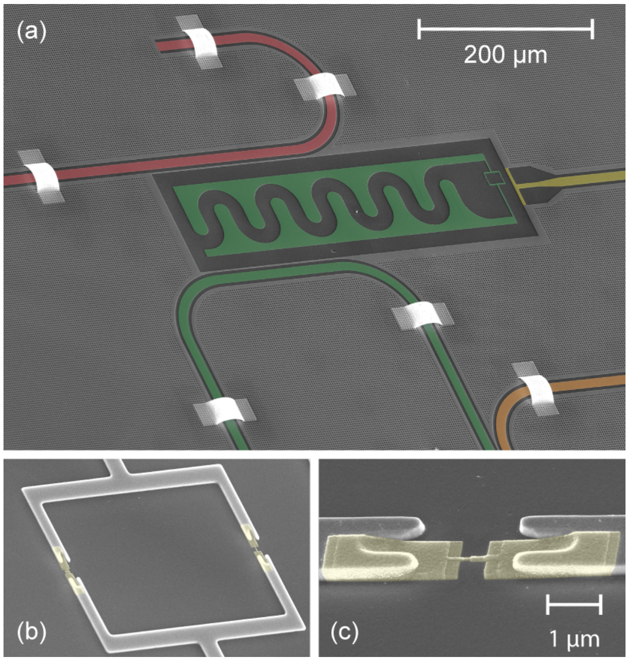

Supplementary Figure 2. SEM images of a sister device with added false colour. (a) Rabi qubit (Q_R) with coupling to the Rabi resonator (R_R, above) and readout resonator (below), showing the centred flux-bias line and displaced SQUID loop. Q_R is coupled to R_R near its shorted end in order to achieve the required small coupling g. (b, c) Josephson junctions are contacted to the NbTiN SQUID loop fingers using small bays to achieve better contact. In (b), it is possible to see the large asymmetry in junction size, with a zoom on the small junction in (c).

Supplementary Note 2: Device fabrication

The device was fabricated using a method similar to that of Ref. 2, but with several specific improvements:

1. The transmon design includes a rounded spacing between the shunt capacitor plates [Supplementary Fig. 2(a)] to avoid the regions of high electric field which can increase sensitivity to interface two-level fluctuators [3].

2. The flux-bias line was centred between the transmon capacitor plates to symmetrise the capacitive coupling with the goal of decoupling the qubits from possible decay-inducing effects of voltage noise fluctuations on the flux-bias lines.

3. As in our previous work [2], the transmon qubits were patterned with niobium titanium nitride (NbTiN) capacitor plates to further reduce susceptibility to noise from two-level fluctuators. Prior to evaporation of the aluminium (Al) junction layers, a short hydrogen-fluoride (HF) dip removed surface oxides to facilitate a good contact between the evaporated Al and NbTiN thin film. To avoid contact problems caused by unwanted etching into the silicon substrate during patterning of the NbTiN, we: 1) optimised the reactive-ion etch (RIE) recipe and duration to minimise the substrate etch and eliminate underetch (under the NbTiN); and 2) introduced a narrow bay in the NbTiN fingers at the contact point to create a softer etch for more reliable contact [Supplementary Fig. 2(c)].

4. The junction development process and double-angle evaporation parameters were optimised to improve the reliability of the very small junction sizes needed for the asymmetric qubit [Supplementary Fig. 2(b)].

Supplementary Note 3: Device operating parameters and qubit performance

Supplementary Figure 3(a) shows the frequencies for the two qubits and three resonators on the device as a function of the applied qubit flux in units of the flux quantum $\Phi_0 = h/2e$, along with the operating points for both qubits during the quantum simulation experiments. Measured device parameters are summarised in Supplementary Table 1. Qubit T_1, $T_{2,\mathrm{echo}}$ and T_2^* decay times are shown as a function of qubit frequency in Supplementary Fig. 3(b,c,d).

At the operating point, the Rabi qubit Q_R was designed to sit below the resonator R_R and be pulsed up into resonance with it to avoid continually crossing the resonator with the Q_R's 1–2 transition during the long flux-pulse sequence. Because of significant protocol times and two operating points, an asymmetric qubit design with two flux-insensitive “sweet” spots was used for Q_R [5], with drive pulses applied at its bottom sweet spot. The first-order flux insensitivity at this point also mitigated some of the impact of rapid, long-range flux-pulsing on the qubit pulse tuning. The maximum and minimum frequencies for Q_R in the final cooldown were 6.670 GHz and 5.451 GHz, respectively.

The asymmetric design also minimised the stringent challenge of targetting the qubit frequency to resonator closely on the scale of the very small coupling frequency. Ideally, the resonator would have been closer to the qubit top sweet spot to maximise phase coherence also during the interaction pulses. However, with the asymmetric design, the reduced flux gradient relaxes this constraint. With an asymmetry parameter of $\alpha = (E_{\mathrm{J,max}} - E_{\mathrm{J,min}})/(E_{\mathrm{J,max}} + E_{\mathrm{J,min}}) \sim 0.68$, the Ramsey time T_2^* for Q_R did not typically drop below a few microseconds, even at the positions with steepest flux gradient.

The asymmetry of Q_R was smaller than targetted, with the result that the bottom sweet spot was also lower in frequency than intended. The ancilla qubit Q_W

Component	Frequency domain		Time domain	
Q_{R}	f_{max}	6.670 GHz	At operating point:	
	f_{min}	5.451 GHz	T_1	20–30 μs
	α (asymmetry)	0.68	$T_{2,\mathrm{echo}}$	30–60 μs
	$E_{\mathrm{C}}/2\pi$	-281 MHz	T_2^*	20–50 μs
	f_{readout}	7.026 GHz		
	$g_{\mathrm{readout}}/2\pi$	43 MHz		
R_{R}	f	6.381 GHz	$T_{1,\mathrm{r}}$	3–4 μs
	$g_{\mathrm{r}}/2\pi$ (to Q_{R})	1.92 MHz	$g_{\mathrm{r}}/2\pi$	1.95 MHz
	χ_{w}/π (to Q_{W})	-1.26 MHz		
Q_{W}	f_{max}	5.653 GHz	At operating point:	
	f_{exp}	5.003 GHz	T_1	30–40 μs
	E_{C}	——	$T_{2,\mathrm{echo}}$	5–7 μs
	f_{readout}	6.940 GHz	T_2^*	1.5–1.8 μs
	$g_{\mathrm{readout}}/2\pi$	42 MHz	At top sweet spot:	
			$T_{2,\mathrm{echo}}$	30–60 μs
			T_2^*	20–50 μs

Supplementary Table 1. **Measured device parameters and qubit and resonator performance.** The coupling strength between Q_{R} and R_{R} was measured both by spectroscopy of the avoided crossing, and time-domain measurement of the vacuum Rabi oscillation frequency. For both qubits, Ramsey sequences measured at the sweet spots exhibited beating consistent with quasiparticle tunnelling [4]. T_2^*s reported here were measured by fitting a decaying double sinusoid to a long, beating Ramsey signal and represents the underlying coherence of the qubits. At the operating point for Q_{W} far from the sweet spot, no beating was observed in the Ramsey measurements.

(a standard symmetric transmon) was therefore operated around 650 MHz below its own maximum-frequency sweet spot of 5.653 GHz. At this operating point, its T_2^* was typically $\gtrsim 1.5$ μs. Because we were able to drive Q_{W} and achieve good photon-sensitive operation at this lower position, we chose not to rapidly tune its frequency up to the sweet spot to perform the photon meter measurements.

To identify the flux operating point that positioned Q_{R} precisely at the bottom sweet spot, we applied the following procedure. We first decoupled the applied DC qubit fluxes, applying the appropriate linear correction to compensate for flux cross-talk. Then, after positioning Q_{W} roughly at its selected operating point, we applied a simple excitation swapping sequence for Q_{R} with R_{R} with fixed swap time (near a full swap) and varying amplitudes of positively and negatively directed pulses. Finally, we varied the applied flux on Q_{R} and identified the operating point as the symmetric flux point where the qubit hit the resonance for positive and negative pulses of equal amplitude. We were able to identify this point to 1 part in 5000. Because the precise choice of operating frequency for Q_{W} was not critical, any slight shift in frequency due to residual DC cross-talk remaining after the flux decoupling measurements was unimportant.

Supplementary Note 4: Calibration of the flux distortions

Implementing the digital Trotterisation of the Rabi model proposed in Ref. [6] required tuning the qubit frequency with a long series of square interaction pulses. To achieve this, it was necessary to compensate for the filtering effects of electronics and microwave components in the line [Supplementary Fig. 1] [7]. One of the particular challenges of an experiment using a long train (up to 10 μs) of very short pulses (10–20 ns) is that the system is sensitive to both short- and long-time pulse distortions. These effects included the intrinsic bandwidth of the AWG and the flux-pulse amplifier, the high-pass characteristics of the bias tee, a range of low-pass effects including the Minicircuits and eccosorb filters and filtering from the skin effect of the coaxial cabling, pulse bounces at impedance mismatches, as well as more intangible effects such as transient decays in step responses. Subject to the system operating in a linear regime (e.g., the AWG operating in a comfortable amplitude range), this could be achieved by applying predistortions to the target fluxing sequence.

Supplementary Figure 4 illustrates the calibration process used in this experiment. Rather than building a single, comprehensive model for all flux distortions, we took a divide-and-conquer approach, applying a series of corrections to compensate individual effects. For processes outside the fridge, we calculated the required compensations by directly measuring the system step response using a fast oscilloscope (R&S RTO1024, 10 Gs/s sampling rate and 2 GHz bandwidth). We applied predistortion corrections sequentially, at each step correcting the longest-time behaviour and zooming in to shorter time scales once the longer-time response is successfully corrected. Once measuring through the fridge, we optimised on the shape of the two-dimensional flux-pulse resonance, the so-called "chevron". Again, we typically focussed initially on correcting the coarse features before zooming in

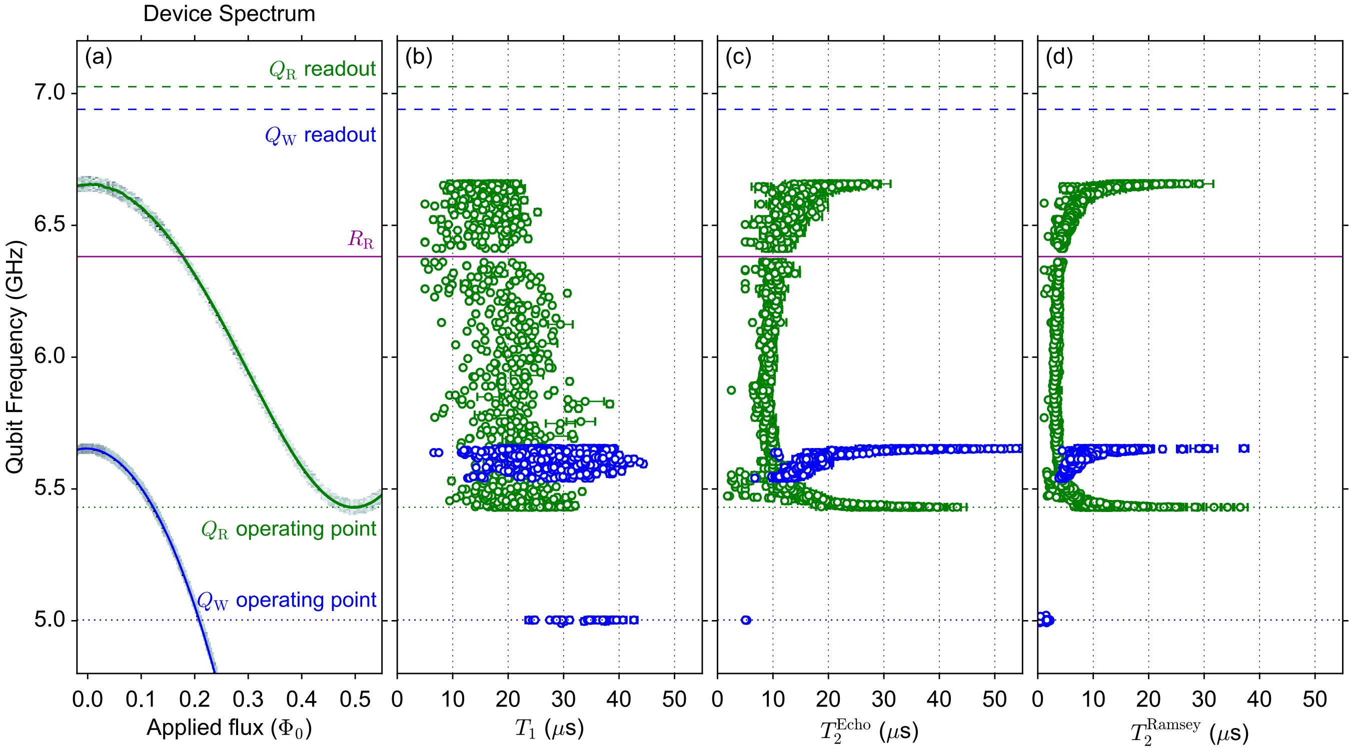

Supplementary Figure 3. Schematic showing measured spectral arrangement for the simulator device and qubit coherence times. (a) Measured data for the 0–1 transition of the Rabi qubit Q_{R} (green curve) and the Wigner qubit Q_{W} (blue curve) are plotted as a function of applied flux in units of Φ_0. Also shown are the frequencies of the Rabi resonator R_{R} (red: $\omega_{\mathrm{r}} = 6.381$GHz) and the readout resonators for Q_{R} (green dashed: ~ 7.03 GHz) and Q_{W} (blue dashed: ~ 6.94 GHz). The operating points of the qubits for the Trotter simulation are given by the green and blue dotted lines for Q_{R} and Q_{W}, respectively. (b, c, d) Time constants measured for Q_{R} (green) and Q_{W} (blue) for (b) T_1, (c) $T_{2,\mathrm{echo}}$ and (d) T_2^*. Note that, at the sweet spots, measured qubit T_2^* times here are limited by slow frequency-switching processes in the qubits such as quasiparticle tunnelling [4].

to finer details.

The procedure we used to calculate the external corrections was:

- Sample a measured step response at a period τ: $x[n] = x(n\tau)$.
- Construct the system impulse response function according to: $h[n] = x[n] - x[n-1]$.
- Construct the system transfer matrix H from $h[n]$ (H is a lower-triangular matrix with $h[j]$ in every position on the jth lower diagonal).
- Invert H to find the transfer matrix of the so-called predistortion kernel and calculate the step response of the predistortion kernel as $Hu[n]$, where $u[n]$ is the discrete Heaviside function. This numerical matrix inversion step limits the length of the step response that can be treated in this way. The sampling period τ is chosen to ensure the sampled step response covers the region of interest.
- Fit the numerically inverted kernel step response using a simple functional form which can then be used to construct a high-resolution predistortion kernel (the impulse response calculated as above from a high-resolution step response). The down-sampling of the step response reduces the fit function dependence on high-frequency effects. For each step, we varied the sampling period to check that the fit parameters were relatively robust to details of the sampling.

Supplementary Figure 4(a) shows the step response from the AWG measured after the home-built flux-pulse amplifier [see Supplementary Fig. 1], with a zoom into the top of the step in (b). In this case, the longest-time response was actually an effectively linear ramp over the long step response. Here, we used a slightly modified procedure to the one above, fitting a linear function directly to the measured step response. Using Laplace transformations, it is possible to show that a step response with a linear ramp, $(1 + \alpha t)\,u(t)$, can be corrected using a predistortion kernel with an exponentially decaying step response $\exp(-\alpha t)\,u(t)$. After this linear correction, we then implemented a series of three corrections with

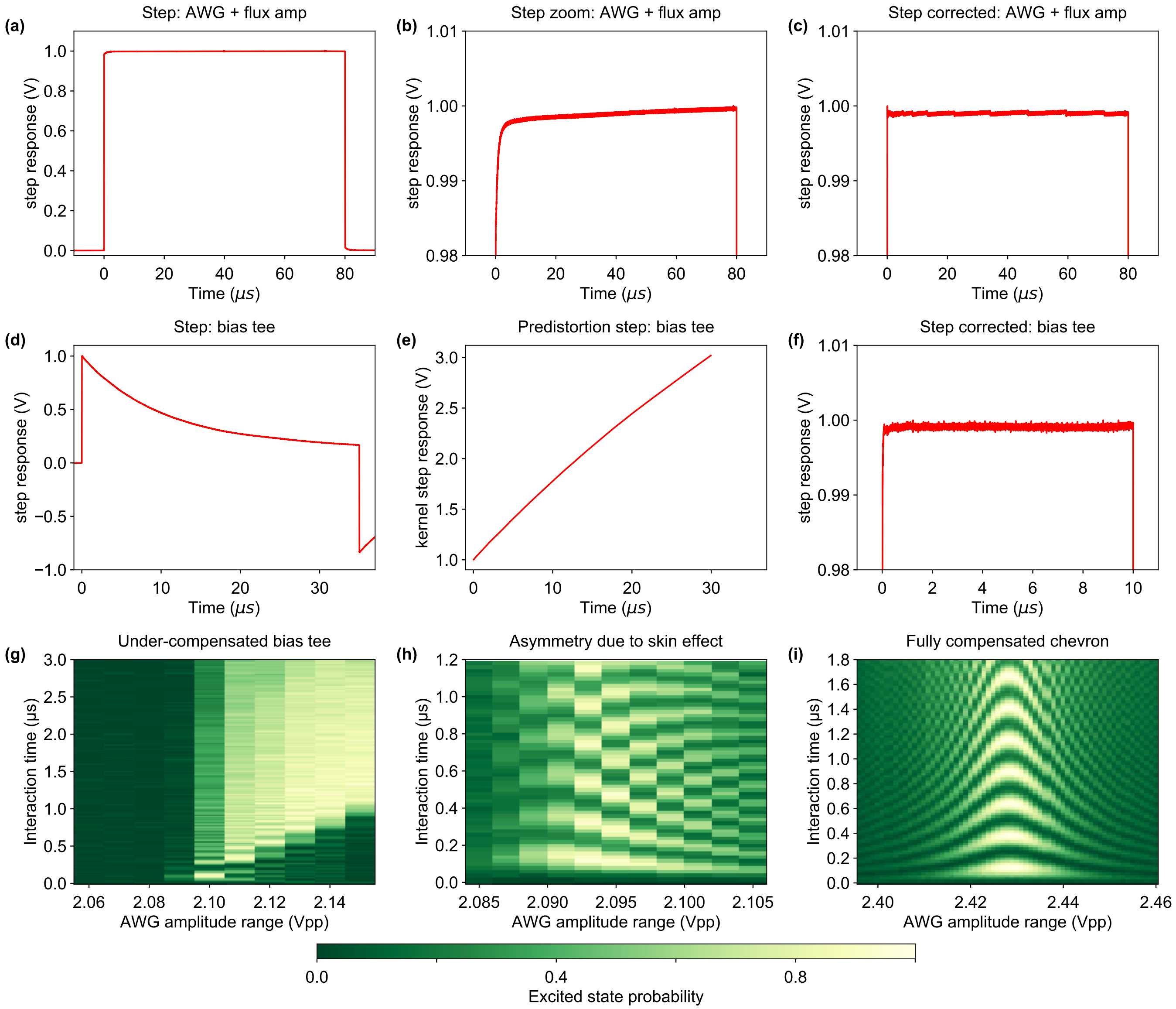

Supplementary Figure 4. **Calibration of the flux distortions.** (a,b) Step response of the amplified AWG flux channel output with a zoom in (b), measured using a fast oscilloscope. (c) Corrected step response achieved using one linear response correction and three exponential decay corrections with parameters (τ, α): (5.1 μs, 0.0012), (670 ns, 0.015) and (520 ns, -0.00037) (see text for details). (d, e) Measured step response (d) and numerically calculated predistortion step response (e) after the bias-tee. (f) Corrected step response achieved using a quadratic bias tee correction (see text for details). (g) Distorted flux "chevron" measured with the corrections applied in (e). (h) Dramatically improved chevron obtained after sweeping one parameter in the bias-tee correction (that corresponding to the standard RC time constant). The asymmetric signature observed here is characteristic of the low-pass filtering effect produced by the skin effect in the coaxial cables. (i) A well-compensated chevron obtained after applying a correction for the skin effect and several more exponential decay corrections with (τ, α): (350 ns, -0.0063), (600 ns, -0.0037), (1500 ns, -0.002), (100 ns, -0.0017) and (30 ns, 0.0036).

"exponential-approach" predistortion step responses of the form $(1 + \alpha \exp(-t/\tau))u(t)$ with τ values between 5 μs and 500 ns (various amplitudes), determined using the above procedure. Supplementary Figure 4(c) shows the corrected step function measured after applying the four initial corrections. The small but distinct sawtooth structure in the otherwise flat step response is due to the vertical resolution of the AWG.

After correcting for distortions from the AWG and flux-pulse amplifier, we measured the step response after the bias tee, at the fridge input. Supplementary Figures 4(d, e) show the measured step response and sampled predistortional kernel step response calculated using the above procedure (with τ = 50 ns). The high-pass

characteristics of a reactive bias tee's RF input naïvely predict a kernel step response with a full initial step followed by a continually increasing linear voltage ramp. From Supplementary Fig. 4(e), however, it is clear that the kernel step response is not completely linear. We instead fit the step response to a quadratic form and proceed as above. The step response measured after compensating for the bias tee is shown in Supplementary Fig. 4(f).

Inside the fridge, we calibrated the flux-pulse predistortions to optimize the shape of the flux chevron [Supplementary Figs 4(g–i)], which probes the excitation-swapping exchange interaction between qubit Q_{R} and resonator R_{R} as a function of flux-pulse amplitude and interaction time. When the qubit is exactly on resonance, the swapping interactions are expected to be slowest and strongest. As it moves off resonance, the oscillations speed up and reduce in amplitude. Interestingly, despite the good performance of the bias-tee correction when measured outside the fridge, the chevron measured with the same corrections [Supplementary Fig. 4(g)] showed a clear ramp in the start of the interaction signal (the lateral skew), consistent with an under-compensated bias tee. We do not understand the cause of this discrepancy, but corrected it empirically by adjusting the linear coefficient of the bias-tee correction. The chevron measured after optimising this correction (final linear coefficient corresponded to a time constant $\tau = 9.7~\mu$s) showed the characteristic asymmetric signature of low-pass filtering from the skin effect [Supplementary Fig. 4(h)]. This was corrected by applying a kernel numerically calculated from a step response of the form $(1-\mathrm{erf}(\alpha_{1\mathrm{GHz}}/21\sqrt{t+1}))\,u(t)$ [8], using $\alpha_{1\mathrm{GHz}} = 1.7$ dB. Finally, we implemented another series of exponential-approach kernels with values of τ between 1500 ns and 30 ns, to achieve the result in Supplementary Fig. 4(i).

Supplementary Note 5: Operating principle and calibration of the photon parity and number meters

Using a number meter based on a Ramsey sequence's sensitivity to qubit frequency and Q_{W}'s dispersive frequency dependence on resonator photon number allows detection of average photon number with controllable sensitivity and dynamic range. When the appropriate wait time between Ramsey pulses is chosen, the same sequence also implements the standard photon parity meter used previously in, e.g., Ref. [9]. We first describe the operating principle of a generic photon meter and then describe the self-consistent calibrations used to tune up both parity and number photon meters. We use a Ramsey-based photon number meter over photon-number-resolved spectroscopy [9, 10] or qubit detection [11], because in our device it proved faster, more flexible, more stable and better calibrated.

Suppose the resonator is in the state $\psi = \sum_j \alpha_j |j\rangle$. To implement the photon meter, we apply a Ramsey pair of $\pi/2$ pulses with pulse separation τ on Q_{W} at a frequency $\Omega_{\mathrm{W}}^{d} = \Omega_{\mathrm{W}}^{0} - d2\chi$, corresponding to the dth photon peak. Different photon-number frequency components accrue different phases during the variable delay between pulses, given by $\theta_j = (j-d)\,2\chi\tau$. By driving first around σ_{x} and then around σ_{y}, the dth photon term ends up on the equator of the Bloch sphere. Measuring the excitation of Q_{W} then gives a measurement probability

$$p_{\mathrm{W}}^{\mathrm{e}} = \sum_j \frac{|\alpha_j|^2}{2}(1+\sin\theta_j). \tag{1}$$

Provided τ is chosen such that θ_j is small for all photon components j present in the photon state,

$$p_{\mathrm{W}}^{\mathrm{e}} = \frac{1}{2}\left(1+\sum_j (j-d)2\chi\tau|\alpha_j|^2\right), \tag{2}$$

$$= \frac{1}{2}\left(1+2\chi\tau(\bar{n}-d)\right). \tag{3}$$

Increasing τ therefore increases the sensitivity of measured probability to average photon number, but decreases the accessible range of photon numbers for which the linearity condition $\sin\theta_j \approx \theta_j$ holds. By contrast, setting $\tau = \pi/2\chi$ ($\theta_j = \pi$; not small), $d = 0$, and driving around $-\sigma_{\mathrm{x}}$ for the second pulse, then implements a standard photon parity measurement. In this condition, even-photon terms return the qubit to the ground state, while odd terms leave the qubit in the excited state.

An accurate calibration of a generic photon meter also requires an accurate calibration of the single-photon dispersive frequency shift 2χ and Q_{W}'s zero-photon frequency (which determines Ω_{W}^{d}). Here, we describe a self-consistent calibration of our photon meters which does not rely on quantities derived from other measurements, such as spectroscopy, and relies primarily on knowing drive-pulse frequencies, probably the most accurate control parameter we have in the experiment. At each stage, we first calibrate Q_{W}'s zero-photon frequency using a standard Ramsey sequence. With the performance of Q_{W} at the operating point (dephasing time $T_2^* \sim 1.5~\mu$s), we routinely achieved frequency accuracy better than 10 kHz.

To calibrate the single-photon dispersive shift [sequence shown in Supplementary Fig. 5(a)], a calibrated SWAP pulse on Q_{R} transfers an excitation into R_{R}, and the resonator photon number is probed via Q_{W}. The single-photon excitation in R_{R} dispersively shifts the frequency of Q_{W} by 2χ. Driving Q_{W} at the calibrated zero-photon frequency around σ_{x} and then σ_{y}, the correct parity condition corresponds to the point where the curve crosses 0.5 excitation probability [Supplementary Fig. 5(d): 383 ns wait time]. This measurement is robust

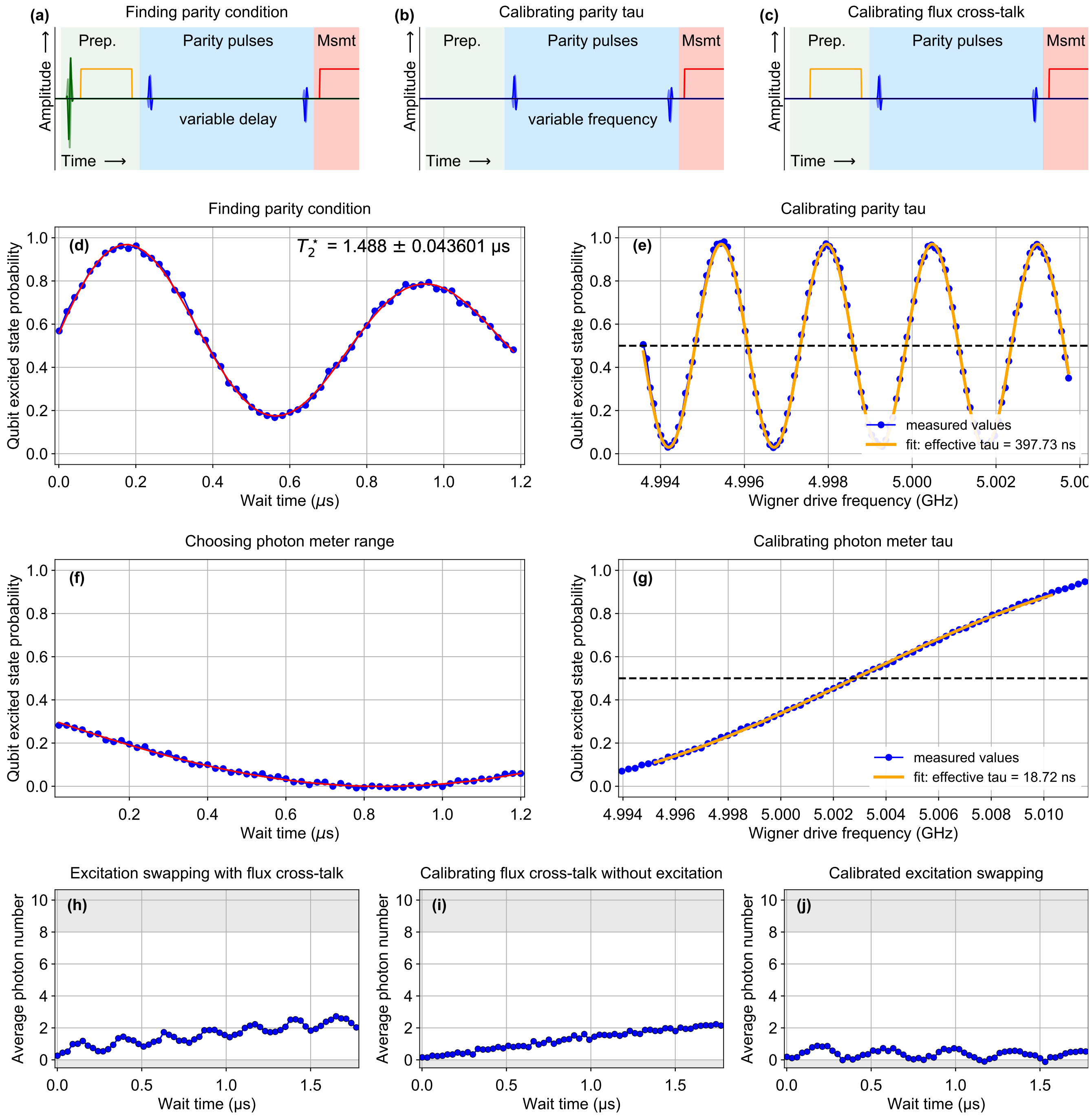

Supplementary Figure 5. **Calibration of the photon meter.** (a–c) Measurement sequences used for calibrating the parity meter, specifically: (a) the dispersive shift of R_{R} on Q_{W}, (b) the effective delay time τ corresponding to a particular pulse separation, and (c) high-frequency flux cross-talk between flux pulses on Q_{R} and the flux offset of Q_{W}. (d) Calibrating the parity condition, identified as the first crossing point of a Ramsey experiment with one photon in the resonator, giving a pulse separation of 383 ns. (e) Calibrating the effective delay time τ for a particular pulse separation. Using parity pulses separated by 383 ns, we calibrated the effective separation τ to be 398 ns, corresponding to a dispersive shift $2\chi/2\pi = -1.26$ MHz. (f) Configuring an average photon number meter for a specific dynamic range of 0–8 photons. Driving at the midpoint of the 0–8 photon frequency range, the Ramsey pulse separation is chosen to lie on the edge of the linear region. For 0–8 photons, we chose to use a separation of 4 ns. (g) Calibrating the photon meter effective τ. Repeating the measurement described in (e), the effective pulse delay for a 4 ns separation was $\sim$ 19 ns. Comparing the oscillation period of the curves in (e) and (g) highlights the different sensitivity of the two photon meters. (h–j) Calibrating high-frequency flux cross-talk. The flux cross-talk is calibrated by measuring the photon meter without loading excitations into the resonator and corrected by adjusting the phase of the second photon meter pulse.

to both the relatively short resonator photon decay time $T_{1,\mathrm{r}} \sim 3.5$ μs and the short dephasing time of Q_W at its operating point ($T_2^* \sim$ 1.5–1.8 μs at ~ -650 MHz detuned from its top sweet spot), because these processes both reduce the visibility of the curve, but not the oscillation period, and therefore do not affect the value of the crossing point. The zero-photon frequency calibration is the main limitation, because that calibration limits the accuracy with which the crossing point represents the correct delay time between $\pi/2$ pulses.

The wait time identified above specifies the time between the end of the first pulse and the beginning of the second required to realise a photon parity measurement, but this does not account for the finite pulse duration. To calibrate the effective value of τ, we fix the pulse separation and sweep the frequency of the Q_W drive generator this time without loading any photons into the resonator [Supplementary Fig. 5(b)]. For a pulse separation of 383 ns, the effective τ is $\sim$ 398 ns [Supplementary Fig. 5(e)]. Note that the difference here is not quite the same as the drive pulse width used in the experiment ($4\sigma = 12$ ns). This value of τ is related to the dispersive shift of R_R on Q_W in the usual way: $\tau = \pi/2\chi$, giving $2\chi/2\pi = -1.26$ MHz. Note that, when used directly as a parity meter, the read-out of Q_W was calibrated using a parity pulse pair either with the usual phase on the second pulse, or a phase shifted by π radians. This accounted for the reduced parity visibility from the short T_2^* of Q_W at its operating point and helped to track any fluctuations in the correct parity extremes as a result of drift in qubit frequency and T_2^*.

Supplementary Figures 2(a, b) show the pulse sequences for two different photon meters used in the experiment, one with the standard Ramsey sequence [calibrations in Supplementary Figs 5(f, g)] and one an unbalanced "echo"-like sequence with an off-centre refocussing pulse (calibrations not shown). The mapping between average photon number and qubit excitation is approximately valid provided the phase advance/delay is less than 30 degrees, which corresponds to a qubit excitation of 0.25. We select the appropriate Ramsey pulse separation by driving the qubit at the frequency corresponding to the mid-point of the desired range (here, the 4-photon position), calculated from the dispersive shift and the calibrated zero-photon frequency, and choosing the separation which gives the target excitation probability of 0.25 [Supplementary Fig. 5(f)], here 4 ns. The effective τ was calibrated, as above, to be $\sim$ 19 ns. Moving to the smaller τ necessary for a higher photon number dynamic range requires frequency refocussing. Ultimately, the main limitation to the range achievable with such a photon meter is set by the bandwidth of the drive pulse.

We used a photon number meter calibrated using the above procedure to follow the excitation-swapping oscillations of a vacuum-Rabi exchange between Q_R and R_R, plotted as a function of the duration of the flux pulse on Q_R [Supplementary Fig. 5(h); sequence in Supplementary Fig. 5(a)]. The drifting baseline results from pulsed flux cross-talk between Q_R and Q_W. To correct this, we repeated the same measurement without initially exciting Q_R in order to avoid exciting photons in R_R [Supplementary Fig. 5(c)]. This curve was compensated by adjusting the drive phase of the second Ramsey pulse in the photon meter (on Q_W), leading to the compensated measurement in Supplementary Fig. 5(j). To maximise the sensitivity of the cross-talk calibration, during the calibration, Q_W can be driven at the zero-photon frequency, which then places the expected "null" measurement result on the equator of the Bloch sphere. A modified version of this procedure can be carried out for all flux-pulse sequences of interest. Note that cross-talk compensation was also necessary to ensure an accurate calibration of the parity condition in Supplementary Fig. 5(d) above.

Supplementary Note 6: Calibration of Wigner tomography

We implement Wigner tomography using the direct method of Ref. 9. After the algorithm part of the pulse sequence [represented in Supplementary Fig. 6(a) by a swap], a 50 ns square pulse applies a coherent displacement to the resonator photon state before the usual parity readout pulses. The phase-sensitive resonator drive tone is created via single-sideband modulation in an IQ mixer. We calibrate the drive frequency and amplitude using the already calibrated photon meter [Supplementary Figs 6(b, c), respectively]. The drive amplitude is calibrated in the middle of the linear range, where we expect the best performance. Supplementary Figure 6(c) illustrates the breakdown of the linear mapping between average photon number and Q_W excitation probability both towards the edge of the linear regime and above the range, as the higher photon components wrap around in phase. In the digital QRM simulation, for phase-sensitive Wigner tomograms (e.g., Figs 3 and 4), it was critical to maintain phase stability between the drives on Q_R and R_R during the measurement. To achieve this, the two microwave generators were synchronised using a 1 GHz reference, with frequencies set as a multiple of the 5 kHz experimental repetition rate.

Supplementary Figure 6 shows one- and two-dimensional Wigner tomograms of a zero-photon (d, e) and one-photon (f, g) state (scaled in terms of photon parity). The maximum visibilities in Supplementary Figs 6(f, g) do not reach the expected values, because these tomograms were measured without an accompanying full set of parity meter calibrations. However, the radial symmetry observed in these tomograms demonstrates the correct behaviour of the coherent resonator drive.

The curves in Supplementary Figs 6(d, g) show fits

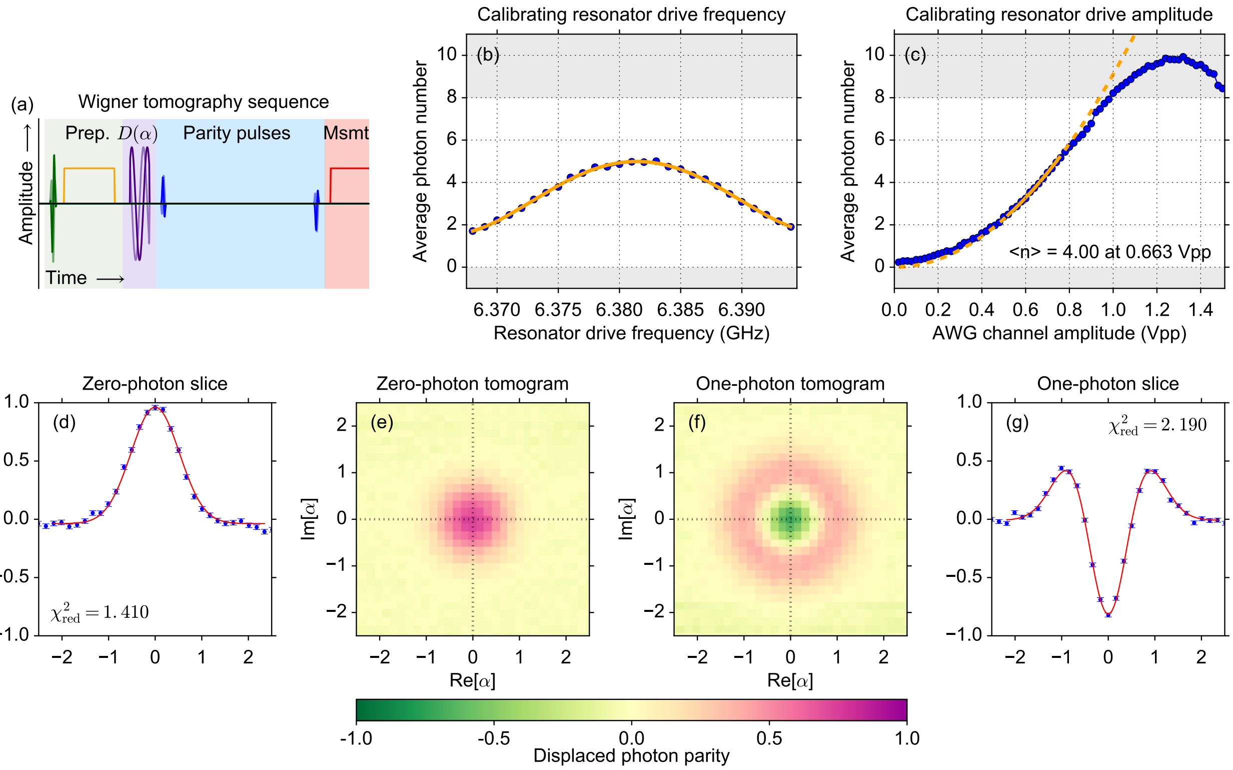

Supplementary Figure 6. **Calibration of Wigner tomography.** (a) Pulse sequence used to make the displaced photon parity measurement which provides a direct measurement of the Wigner function at a particular position in phase space. (b) This plot shows the response of $R_{\rm R}$ to the drive pulse as a function of drive frequency, as recorded by the $Q_{\rm W}$ photon meter, centred at 6.3814 GHz, with a FWHM of $\sim$ 21 MHz, in reasonable agreement with the 18 MHz expected for a 50 ns square pulse. (c) The pulse displacement amplitude is also calibrated using a low-dynamic-range photon meter with a linear range of 0–8 photons. We fit the data in the centre of the linear range, where the photon meter mapping is most accurate, with a function of the form $\langle n \rangle = k_{\rm A} A^2$, finding $k_{\rm A} = 9.10$. (e, f) Measured direct Wigner tomograms of zero-photon (e) and one-photon (f) states (one-photon state prepared using a calibrated SWAP pulse between $Q_{\rm R}$ and $R_{\rm R}$). (d, g) Direct Wigner tomogram slices of zero-photon (d) and one-photon (g) states measured using the full parity meter calibrations.

to the data of a classical mixture of zero-photon and one-photon Wigner function cross-sections, with a free x-axis scaling parameter has been included in the fits. These fits demonstrate that the measured tomograms agree well with theoretical expectations, subject to an x-axis scaling error of $\sim$ 5%. That is, the fits indicate that the amplitude calibrations result in a small systematic overestimate in displacement by 5%. This also agrees with two-dimensional double Gaussian fits of individual frames of the unconditional Wigner movie in Fig. 3(a) of the main text, which give an average Gaussian width $\bar{\sigma} = 0.526 \pm 0.003$, compared with the expected value of 0.5.

Supplementary Note 7: Analog vs Digital Jaynes-Cummings Dynamics

Simple modelling of the Trotterised version of the full Rabi model shows that high-quality simulations require both slow dynamics and short Trotter steps (i.e., fast flux pulsing). Such an experiment is sensitive to both short-time and long-time effects in the flux-pulse shaping. A simpler experiment which verifies the performance of this flux pulsing is to implement a digital simulation of the standard Jaynes-Cummings (JC) interaction underlying the standard excitation-swapping experiments demonstrated with single flux pulses [Supplementary Fig. 4].

In the standard continuous-wave (single-pulse) version of a JC excitation-swapping interaction, resonance between the qubit and resonator frequencies gives rise to maximum visibility oscillations of the excitation moving between the two components. When detuned, the different phases accrued by the qubit and resonator during the interaction decrease the oscillation visibility, while increasing the oscillation frequency. This gives rise to the characteristic shape of the flux chevron. Significant care is required, however, to accurately reproduce the (analog) JC interaction with a digital pulse train.

Supplementary Figures 7(a, b) show analog and dig-

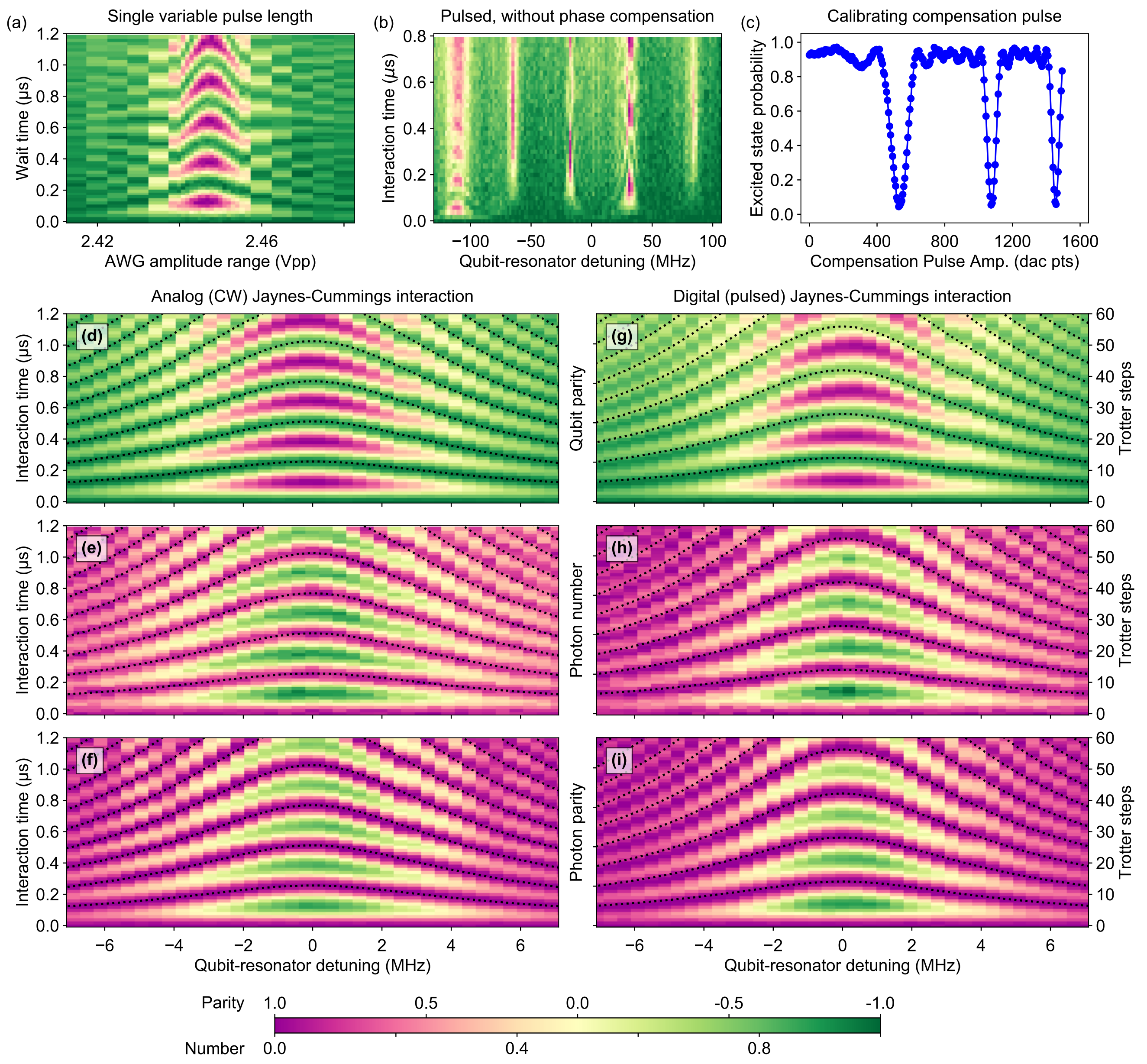

Supplementary Figure 7. **Comparison of analog and digital versions of a Jaynes-Cummings interaction.** (a) Standard analog JC chevron showing the resonant excitation swapping between qubit and resonator after the qubit is initialised in the excited state as a function of flux-pulse amplitude (x axis) and duration (y axis) (qubit-resonator detuning and interaction time, respectively). The x-axis location of the chevron ($\sim$ 2.445 Vpp) therefore defines the qubit-resonator on-resonance condition. (b) Digital JC chevron (measured under otherwise identical conditions) using a pulse duration of 20 ns showing a series of equally spaced resonances with different apparent interaction strengths. (c) We scan amplitude of a 5 ns compensation flux pulse to identify the value which enforces that the digital chevron is centred around the natural resonance position. (d-f) Standard analog and (g-i) digital JC chevrons measured by probing: (d, g) the excited state probability for Q_{R}, (e, h) the average photon number in R_{R} (linear range 0–2 photons), and (f, i) the photon parity of R_{R}.

ital versions of the JC interaction (viewed through the qubit excitation) under otherwise identical conditions. The digital chevron shows a series of resonances which do not appear in analog measurements (not shown), and there is also no chevron visible at the natural resonance condition around 2.45 Vpp.

The new features relate to the extra "interaction off" times in the digital version. The regular spacing between neighbouring satellite resonances is around 50 MHz (after converting AWG amplitude to qubit frequency), which is the inverse pulse duration. During the interaction time, the qubit-resonator relative phase evolves as expected. However, in the "off" time between interaction pulses, the qubit accrues phase at a different rate, and will hence

not have the required phase at the beginning of the next pulse for the interaction to pick up where it left off at the end of the previous pulse. Therefore, the necessary condition for observing a chevron feature at exactly the position of the natural resonance is that the qubit phase accrued (relative to the resonator) during the "off" time should be a multiple of 2π. The observation of multiple satellite resonances is a form of digital aliasing, where the interaction will build up constructively from pulse to pulse provided the relative phase accrued between qubit and resonator during the "on" time of the pulse again differs only by an integer multiple of 2π. However, this is an aliasing of the dynamics itself, not just an aliasing of the measurement, which could also occur in natural continuous-wave (CW) chevrons and would never lead to the observation of extra satellite peaks.

This pulsed interaction can also be viewed as a Trotterised simulation of the CW interaction. While successive interaction pulses obviously commute with each other, they do not necessarily commute with the "off" pulses. The condition on qubit-resonator phase during the "off" pulse can be understood as the condition where the Trotter error vanishes, because the Hamiltonian term resulting from the qubit detuning coincides with the identity. The satellites arise because the phase contribution from the qubit detuning in the "on" pulse is identical if the frequency change matches a multiple of 2π phase.

To compensate for the phase error accrued in the qubit during the "off" pulses, we apply a 5 ns compensation flux pulse between interaction pulses. Using the flux-pulse amplitude which corresponds to the centre of the CW chevron, the amplitude of the compensation pulse was swept to identify the correct compensation point. In this way, very good agreement was achieved between the digital JC dynamics and the traditional analog version [Supplementary Fig. 7(d–i)]. The main differences are a slightly reduced visibility because of the increased experiment time, and a slighly lower effective coupling frequency ($g/2\pi \sim 1.8$ MHz, instead of ~ 1.95 MHz). The latter most likely arises from residual short-time pulse imperfections which do not contribute significantly to the long interactions in the analog form.

Supplementary Note 8: Trotter simulation with excited and ground initial states

In the degenerate-qubit case, when understood in terms of the cavity trajectories in phase space, it is clear that the structure of the expected quantum Rabi dynamics at ultrastrong coupling (USC) or deep-strong coupling (DSC) should not depend on whether the qubit starts in the ground or excited state. This contrasts with the JC interaction, where the $|g, 0\rangle$ state is decoupled from the rest of the system and the system will only undergo nontrivial dynamics if an initial excitation is loaded in the system. Indeed, in a natural USC/DSC system, if it were possible to turn the coupling on and off rapidly, it would be extremely interesting to watch an uncoupled-system ground state evolve into a state with excitations in the qubit and cavity. In this digital simulation, however, this is less satisfying, since the protocol in any case involves regularly injecting excitation into the system in the form of qubit flipping pulses. Most of the results reported here therefore take the more conservative position of initialising the system with an excitation, with the motivation that observing a difference between the simulated dynamics and what would be expected in a weak-coupling scenario could then only result from the simulated counter-rotating terms. Although there were some stability issues during the measurement with ground-state initialisation, there is nevertheless extremely good agreement between the two cases, for example with the timing of the revivals in both cases agreeing with the theoretical predictions. For this particular measurement of ground-state initialisation, qubit revivals are observed even out to $r \equiv g^{\mathrm{R}}/\omega_{\mathrm{q}}^{\mathrm{R}} \sim 0.9$.

Supplementary Note 9: Trotterisation performance vs Trotter order

As discussed already, initial modelling of a Trotterised Rabi simulation showed that unusually low qubit-resonator coupling between Q_{R} and R_{R} was required to be able to achieve reasonable simulation fidelities given the hard bandwidth limitations of flux-based fast frequency tuning. This, however, required longer experimental times for the simulations, which in turn placed significant constraints on qubit and resonator coherence. Indeed, the shorter-than-anticipated resonator coherence time proved to be the biggest limitation. As a result, it was critical to use all available measures to minimize the Trotter error in our simulations, given the limits on the shortest achievable Trotter step sizes.

The accuracy of the Trotter approximation is set by the amount of non-commutativity between different components in the step [12]. While first-order Trotterisations [$\exp(A+B) \approx \exp(A)\exp(B)$] lead to Trotter errors that scale with single commutators (quadratically with simulation time), higher-order Trotterisations can be used to eliminate lower orders of Trotter error. For example, the symmetry of a second-order Trotterisation [$\exp(A+B) \approx \exp(A/2)\exp(B)\exp(A/2)$] ensures that first-order error terms (related to single commutators) cancel, pushing the largest Trotter error terms out to third order in simulation time. For two-part Hamiltonians, however, second-order Trotterisation in practice only involves modifying the pulses in the first and last Trotter steps. All the results in the main text were obtained using a second-order Trotterisation. The plots in

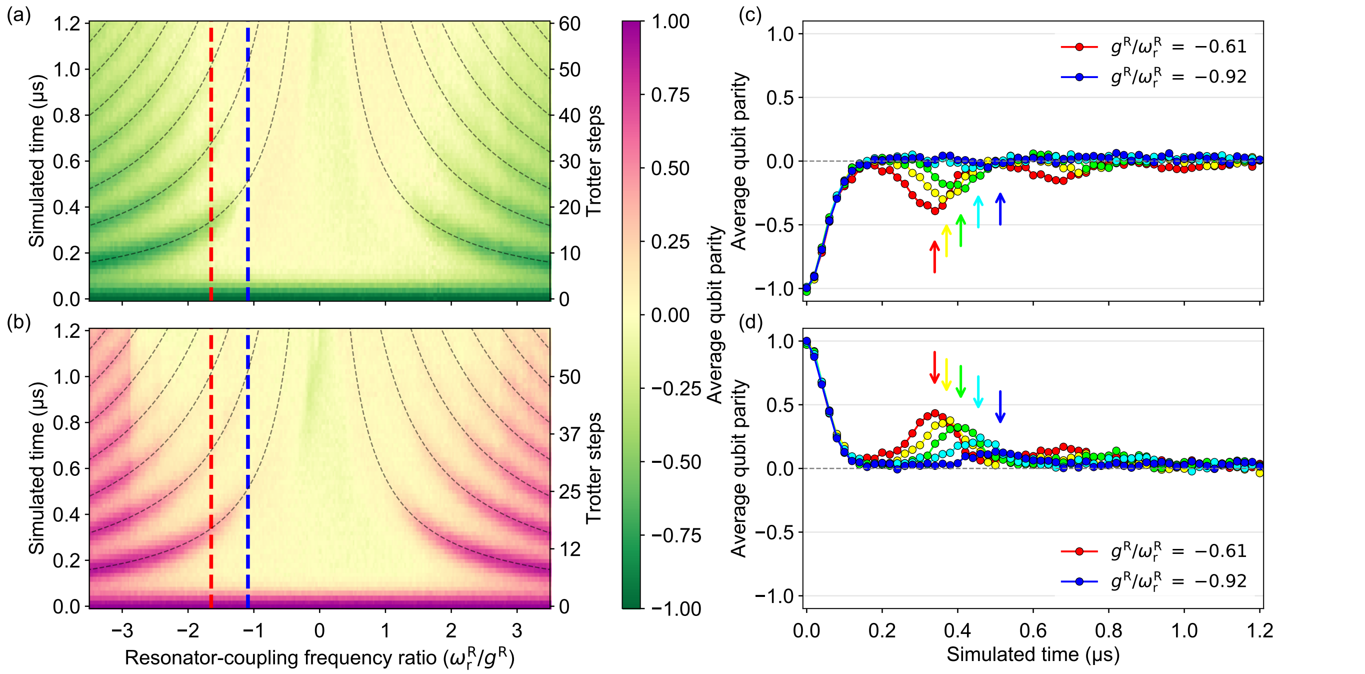

Supplementary Figure 8. **Comparison of average qubit parities for simulations with different initial states.** The plots show simulated qubit parity dynamics when initialising in the excited (top) versus the ground state (bottom). (a, b) These plots directly verify the symmetrical behaviour of the simulated Rabi model. (c,d) Line slices are plotted at evenly spaced frequencies between the red and blue dashed lines in (a, b). Arrows in (c, d) show the expected time for the first revival.

Supplementary Fig. 9 illustrate that this was absolutely critical in order to extend the simulations into the DSC regime. The first-order and second-order Trotterisation agree reasonably well at $r < 0.5$, but behave fundamental differently at the higher values. The first-order simulation starts to show qualitatively different behaviour for relative coupling strengths $r \gtrsim 0.5$. In particular, only in the second-order case are the characteristic plateaus and revivals of the DSC regime observable.

Supplementary Note 10: Trotterisation performance vs Trotter step size

As illustrated in Supplementary Fig. 9, the effects of Trotter error are most visible in the high r regimes, which is reasonable, considering that for low r, the Rabi model is well approximated by the JC model where the excitation-nonconserving terms (non-commuting with the excitation-conserving terms) do not play a significant role. This was also visible when studying the performance of the simulation as a function of the Trotter step size.

Measurements and numerical simulations show significant reduction in Trotter error as the number of Trotter steps over 1.2 μs increased from 24 to 60. The Trotter error shows up in two ways, namely the central features departing from the expected plateaus, and a tendency for the dynamical landscape to "break apart", even out into the lower coupling regimes. In the measured results and the simulation with decay, the fine details do not appear as strongly, but the effect appears to wash out the oscillation dynamics more rapidly. Only at the smallest step size are these effects absent from the measured results, and in the ideal simulations (without decoherence) there are even then central features which only disappear at a still smaller 10 ns step size. The measured results agree very closely with the numerical Trotter dynamics which include only the effect of photon decay, again highlighting that the primary limiting factor in our experiments was $T_{1,\mathrm{r}}$. It is clear from these results that moving towards the smallest possible Trotter steps will be a key challenge for reaching quantum supremacy in complex quantum simulations.

Supplementary Note 11: Qubit entropy dynamics

In the Rabi model, as the resonator states separate, the qubit-resonator entanglement causes the reduced qubit state to collapse towards the maximally mixed state. A revival occurs in the qubit purity only if the underlying entanglement is still present when the resonator states re-coalesce at the origin in phase space. While many possible uninteresting effects may cause an initial collapse in qubit purity, a revival in purity is a signature of entan-

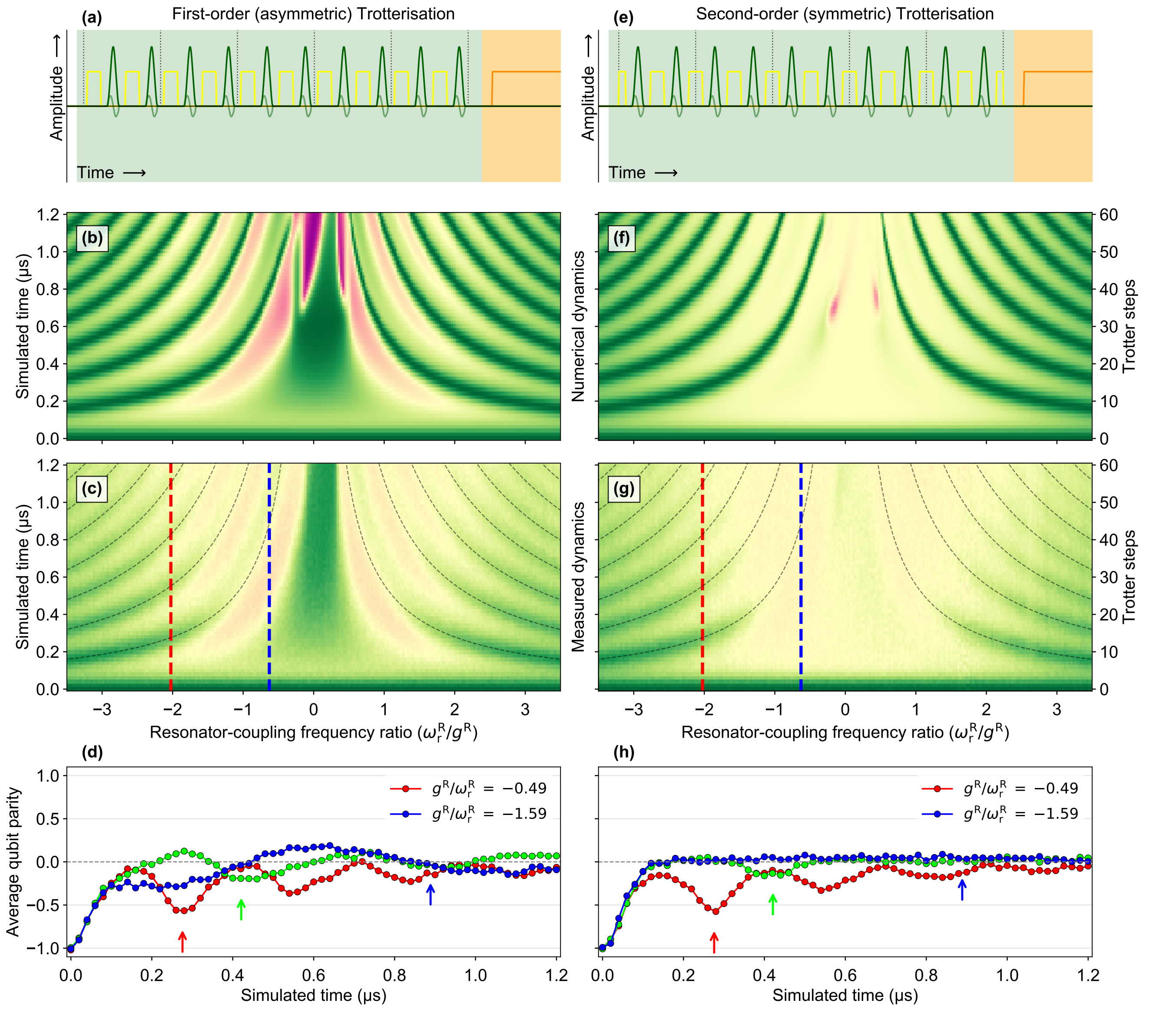

Supplementary Figure 9. **Comparison of simulation performance for different orders of Trotterisation.** Results shown for asymmetric, first-order (a–d) and symmetric, second-order (e–h) Trotterisation. (a, e) Pulse sequences for the first-order (a) and second-order (e) Trotterisation. (b, f) Numerical simulations of the Trotterised Rabi model for the ideal case with no decay. Note that the sharp features in the centre of the plots (DSC regime) are not artifacts of the numerics, but Trotter error related to the 20 ns step size (these features disappear for 10 ns pulses). (c, g) Experimental quantum simulations for first-order (c) and second-order (g) Trotterisation, showing very good agreement with the numerical results in (b, f). (d, h) Vertical line slices are plotted for evenly spaced resonator frequencies between the red and blue dashed lines in plots (c) and (g).

glement with another system, in this case the resonator. After each Trotter step, a tomographically complete set of measurements on Q_R was used to reconstruct its reduced state using maximum-likelihood tomography. We use the von Neumann entropy to characterise the purity of the reduced qubit state and observe revivals in qubit purity out to $r > 0.8$ [Supplementary Fig. 11(a)], consistent with the observed revivals in qubit parity. While the observed revivals shown in the slices [Supplementary Fig. 11(b)] appear smaller than the qubit parity revivals, in fact this is deceiving, resulting from the fact that purity (as with other entropy measures) is a quadratic function of the qubit population difference. The inset shows that the background noise of this signal is small and that the revivals are quite distinct. Moreover, plotting an appropriate square root of the entropy (not shown) shows that the revivals are consistent with the qubit parity case.

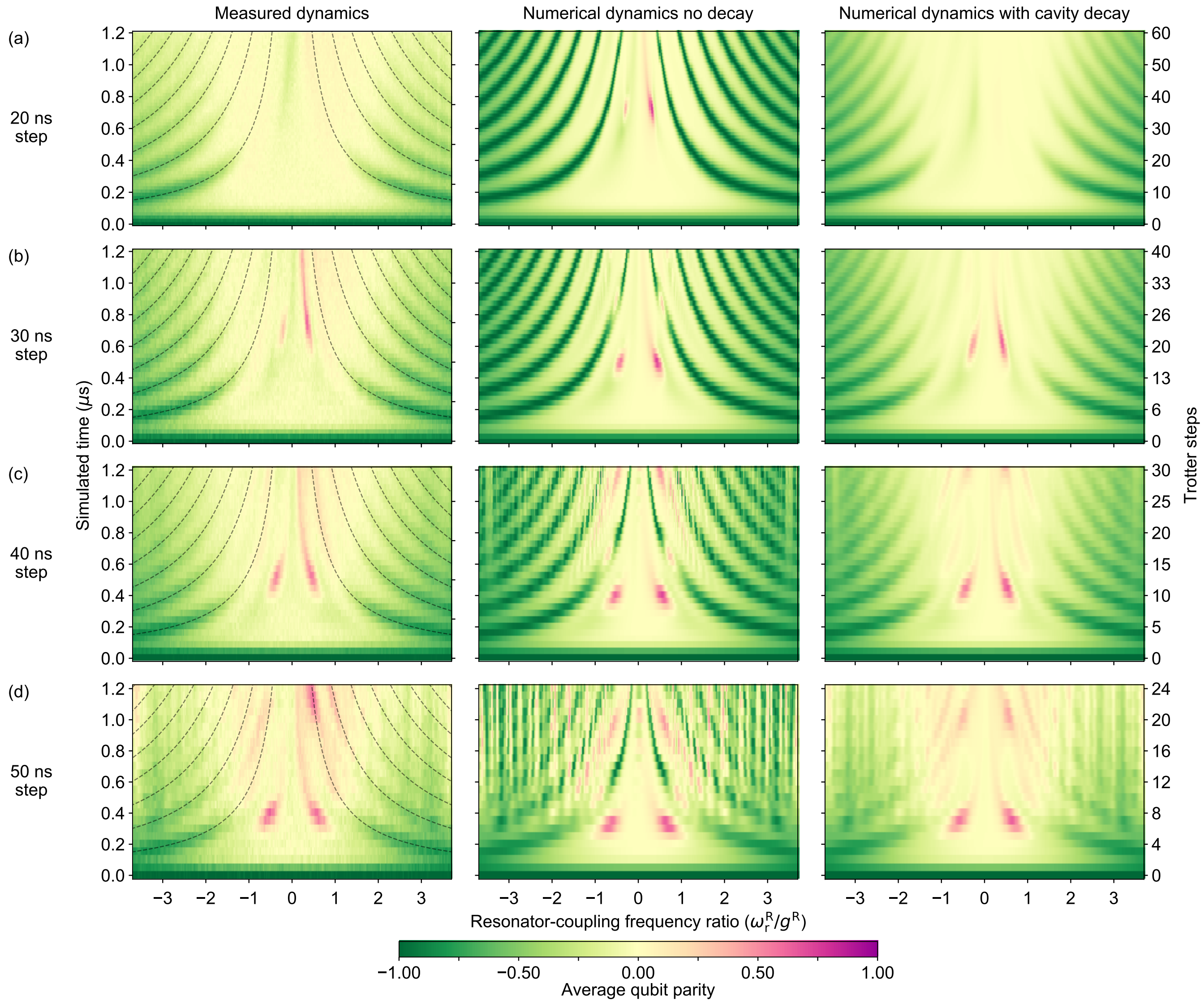

Supplementary Figure 10. **Comparison of simulation performance for various Trotter step sizes**. The results show measurements (left), numerical simulations with no decay (middle) and numerical simulations with the measured $T_{1,\mathrm{r}} = 3.5\ \mu$s: (a) 20 ns steps (60 Trotter steps), (b) 30 ns steps (40 Trotter steps), (c) 40 ns steps (30 Trotter steps) and (d) 50 ns steps (24 Trotter steps).

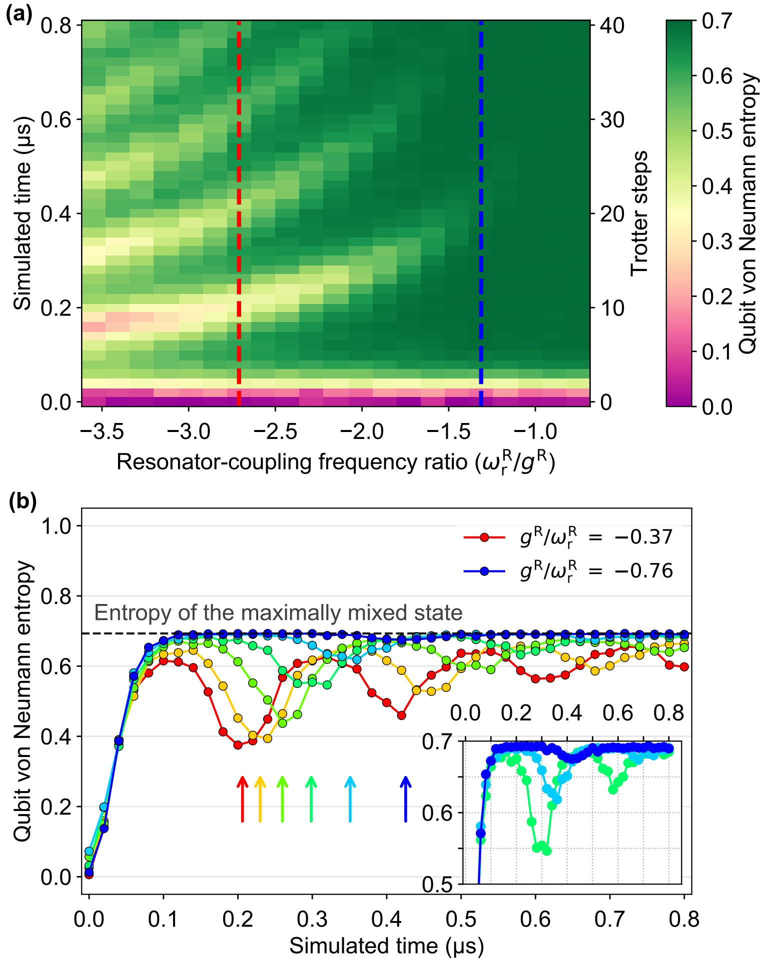

Supplementary Figure 11. **Measured entropy dynamics of the qubit during the quantum simulation.** Entropy is calculated from tomographic reconstructions of the reduced state of qubit Q_R as a function of simulation time and relative resonator-coupling frequency. (a) Image plot showing the dynamics of qubit quantum von Neumann entropy over different USC and DSC coupling regimes. (b) Line slices are plotted at evenly spaced frequencies between the blue and red dashed lines. Inset: Zoom showing revivals.

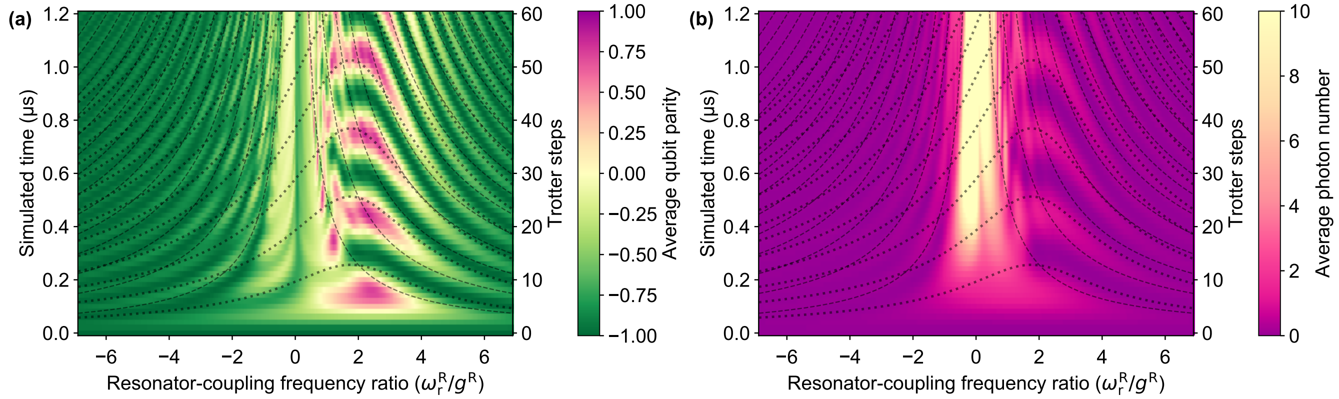

Supplementary Figure 12. Ideal Rabi dynamics for the nondegenerate-qubit case with $g^{\mathrm{R}}/\omega_{\mathrm{q}}^{\mathrm{R}} \sim 0.48$ showing the standard Jaynes-Cummings exchange dynamics emerging from the DSC Rabi dynamics when $\omega_{\mathrm{q}}^{\mathrm{R}}$ becomes significantly larger than g^{R}. (a) Average qubit parity. (b) Average photon number. (Colour scale bar truncated to show details at low photon number.) Expected revival times for pure, degenerate-qubit QRM dynamics (dashed curves) are compared with expected exchange oscillation periods for a pure nondegenerate-qubit Jaynes-Cummings interaction. The colour scale range was chosen to provide visible detail in low-photon regimes (maximum photon number reached in saturated central region $\sim$ 100 photons).

Supplementary References

[1] Barends, R. *et al.*, Minimizing quasiparticle generation from stray infrared light in superconducting quantum circuits. *Appl. Phys. Lett.* **99**, 113507 (2011).

[2] Asaad, S. *et al.*, Independent, extensible control of same-frequency superconducting qubits by selective broadcasting. *npj Quantum Inf.* **2**, 16029 (2016).

[3] Gambetta, J. *et al.*, Investigating surface loss effects in superconducting transmon qubits. *IEEE T. Appl. Supercon.* **27**, 1700205 (2017).

[4] Ristè, D. *et al.*, Millisecond charge-parity fluctuations and induced decoherence in a superconducting transmon qubit. *Nat. Commun.* **4**, 1913 (2013).

[5] Koch, J. *et al.*, Charge-insensitive qubit design derived from the Cooper pair box. *Phys. Rev. A* **76**, 042319 (2007).

[6] Mezzacapo, A. *et al.*, Digital quantum rabi and dicke models in superconducting circuits. *Sci. Rep.* **4**, 7482 (2014).

[7] Johnson, B.R., PhD Dissertation, Yale University (2010).

[8] Wigington, R. & Nahman, N., Transient analysis of coaxial cables considering skin effect. *Proc. IRE* **45**, 166–174 (1957).

[9] Vlastakis, B. *et al.*, Deterministically encoding quantum information using 100-photon Schrödinger cat states. *Science* **342**, 607–610 (2013).

[10] Kirchmair, G. *et al.*, Observation of quantum state collapse and revival due to the single-photon Kerr effect. *Nature* **495**, 205–209 (2013).

[11] Juliusson, K. *et al.*, Manipulating fock states of a harmonic oscillator while preserving its linearity. *Phys. Rev. A* **94**, 063861 (2016).

[12] Lloyd, S., Universal quantum simulators. *Science* **273**, 1073–1078 (1996).
