# Supplementary material for: Experimentally simulating the dynamics of quantum light and matter at deep-strong coupling
Source: Nat Commun. 2017 Nov 23;8:1715. doi: 10.1038/s41467-017-01061-x (PMC5700074; doi:10.1038/s41467-017-01061-x)
Supplement: Supplementary file 2 — Description of Additional Supplementary Files [file 41467_2017_1061_MOESM2_ESM.pdf]

Description of Additional Supplementary Files

File Name: Supplementary Movie 1
Description: **Photon dynamics in phase space in the DSC regime (degenerate-qubit case) from maximum-likelihood Wigner tomography.** (a) Movie, measured over ~ 40 hours, showing the phase-space evolution of the resonator reduced state for $g^{\mathrm{R}}/\omega_{\mathrm{r}}^{\mathrm{R}} \sim 0.9$ (frames labelled by Trotter step n and simulated time). Plotted tomograms are maximum-likelihood reconstructions of direct Wigner tomography measured data with a systematic phase correction [see Methods]. Clearly observed are the creation of two well-resolved, rotating peaks and their subsequent re-coalescence, which are characteristic signatures of DSC dynamics. The final frame shows the full trajectories determined from 2D double-Gaussian fits to the raw data. Deviation from the ideal circular trajectories (orange curves) arises from photon decay. The measured trajectory shows excellent agreement with a numerical Trotter simulation at $g^{\mathrm{R}}/(2\pi)$ = 1.79 MHz which includes resonator $T_{1,\mathrm{r}}$ = 3.5 μs (green curves).
